# Supplementary figures and images for: The tetraspanin TSPAN5 regulates AMPAR exocytosis by interacting with the AP4 complex
Source: eLife. 2023 Feb 16;12:e76425. doi: 10.7554/eLife.76425 (PMC9934860; doi:10.7554/eLife.76425)

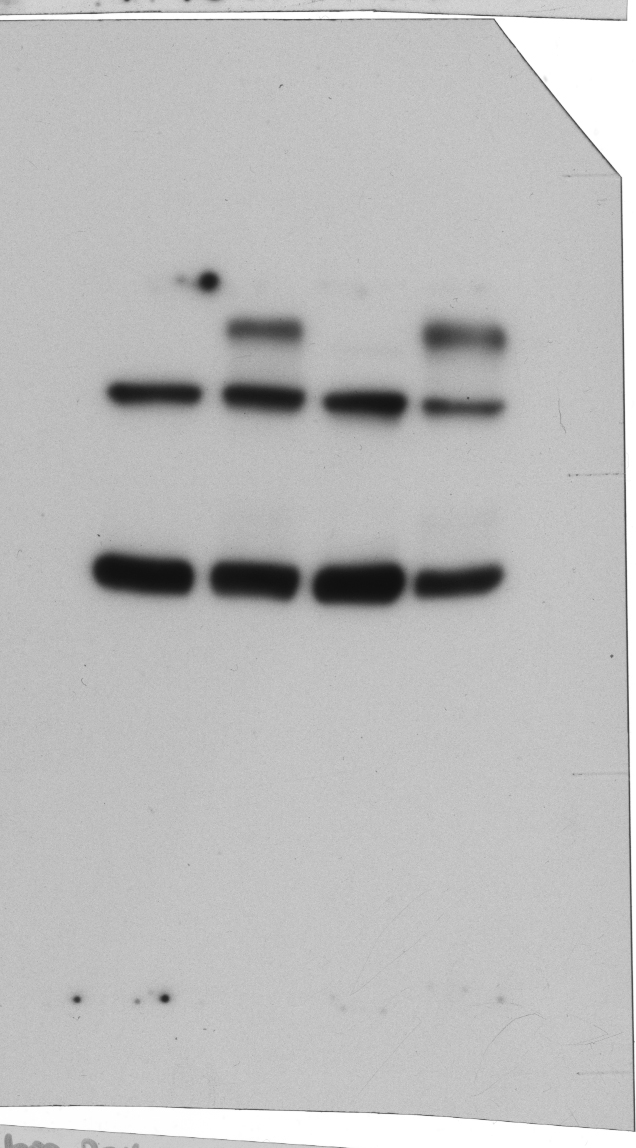

Supplement: Figure 1—source data 2. [file elife-76425-fig1-data2.zip › Figure 1 - source data 2/Figure 1A - TfR blot raw image.jpg]

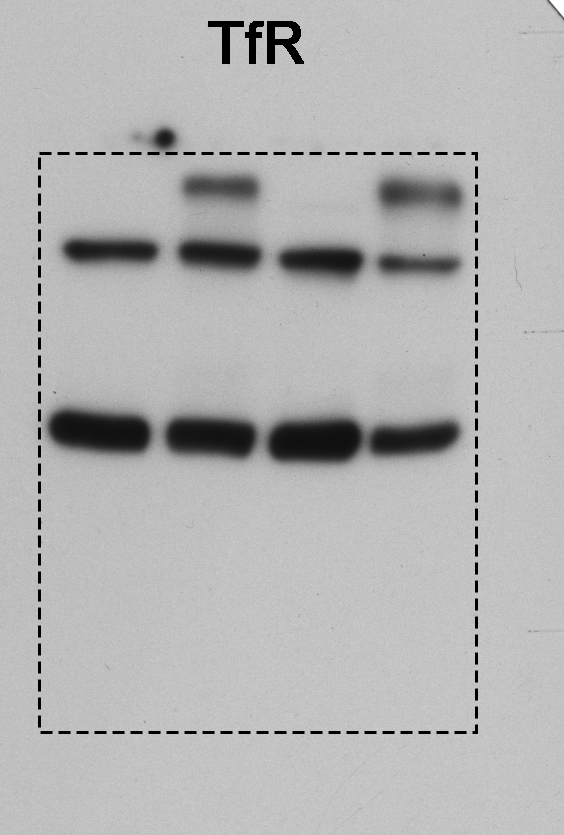

Supplement: Figure 1—source data 2. [file elife-76425-fig1-data2.zip › Figure 1 - source data 2/Figure 1A - TfR blot with cropped area.jpg]

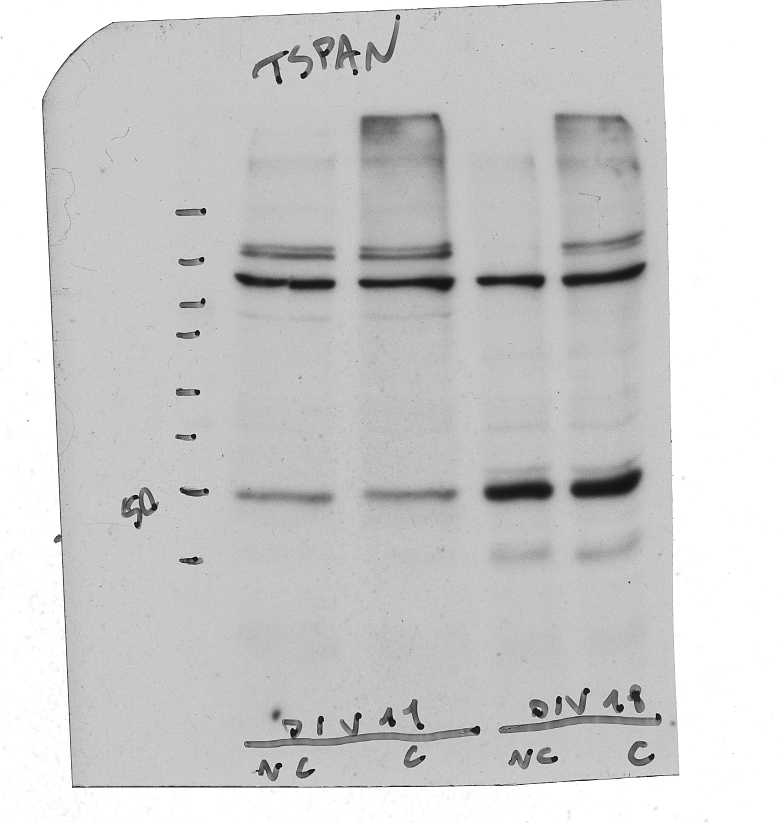

Supplement: Figure 1—source data 2. [file elife-76425-fig1-data2.zip › Figure 1 - source data 2/Figure 1A - TSPAN5 blot raw image.jpg]

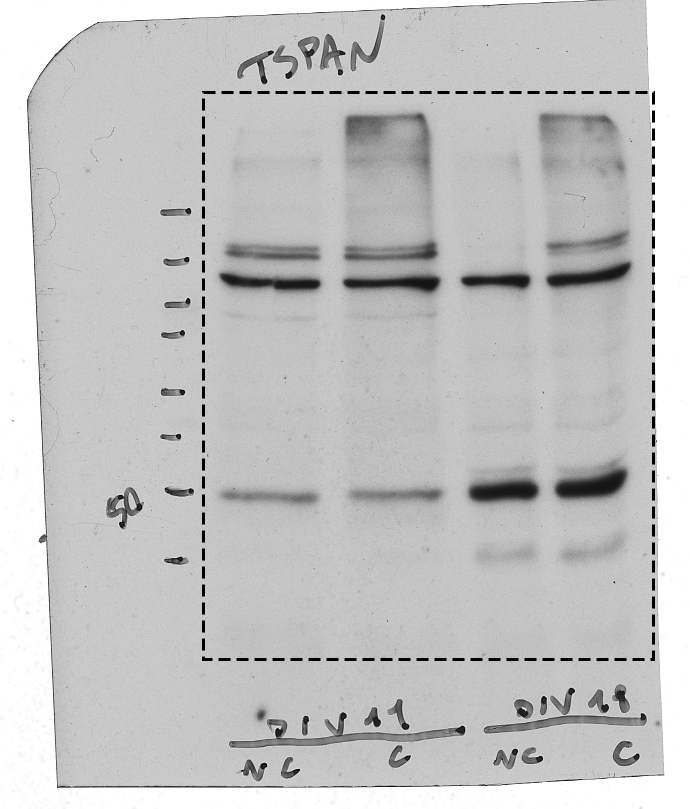

Supplement: Figure 1—source data 2. [file elife-76425-fig1-data2.zip › Figure 1 - source data 2/Figure 1A - TSPAN5 blot with cropped area.jpg]

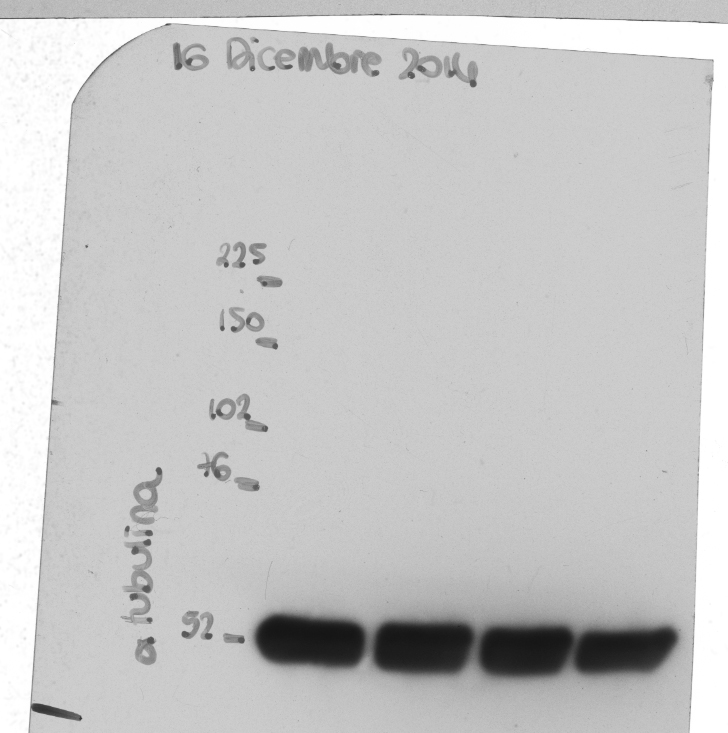

Supplement: Figure 1—source data 2. [file elife-76425-fig1-data2.zip › Figure 1 - source data 2/Figure 1A - Tubulin blot raw image.jpg]

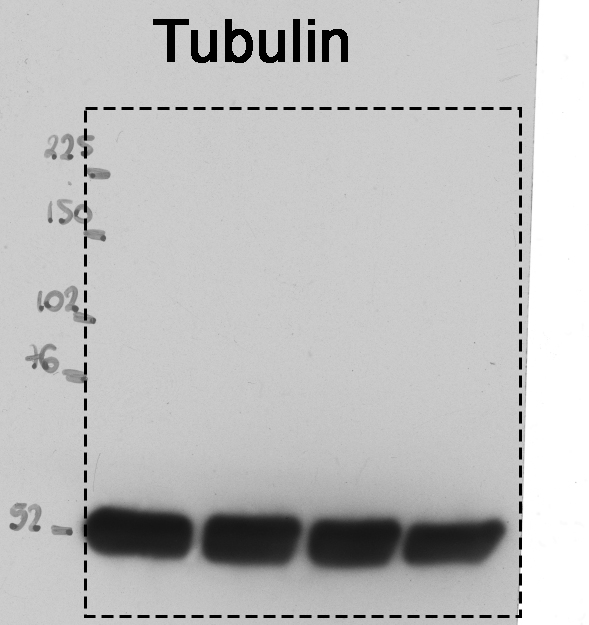

Supplement: Figure 1—source data 2. [file elife-76425-fig1-data2.zip › Figure 1 - source data 2/Figure 1A - Tubulin blot with cropped area.jpg]

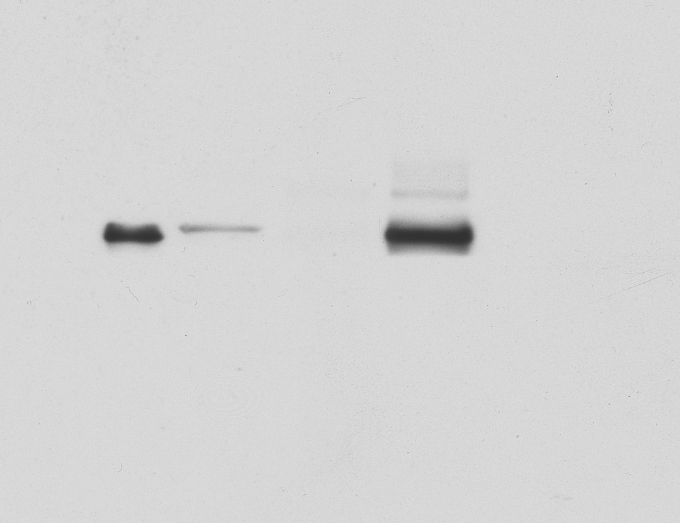

Supplement: Figure 2—source data 2. [file elife-76425-fig2-data2.zip › Figure 2 - source data 2/Figure 2A - AP4E blot raw image.jpg]

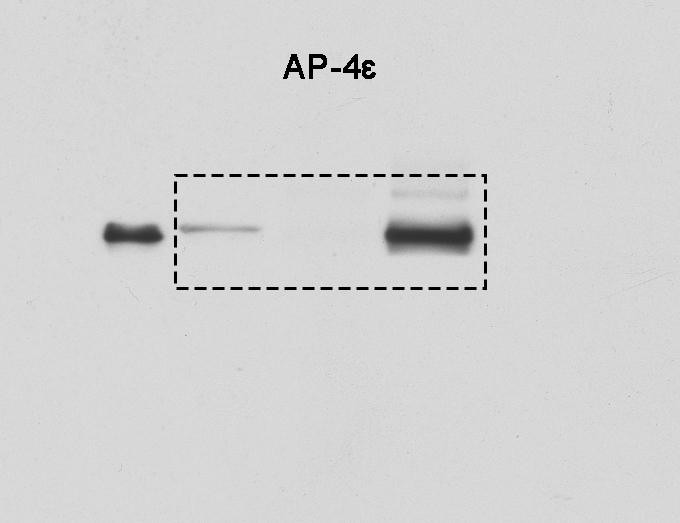

Supplement: Figure 2—source data 2. [file elife-76425-fig2-data2.zip › Figure 2 - source data 2/Figure 2A - AP4E blot with cropped area.jpg]

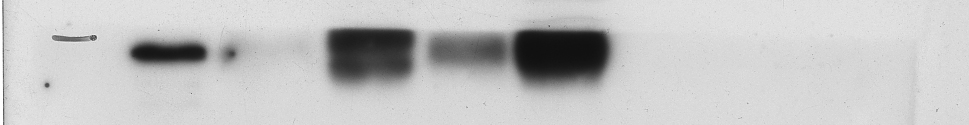

Supplement: Figure 2—source data 2. [file elife-76425-fig2-data2.zip › Figure 2 - source data 2/Figure 2B - AP4 sigma blot raw image.jpg]

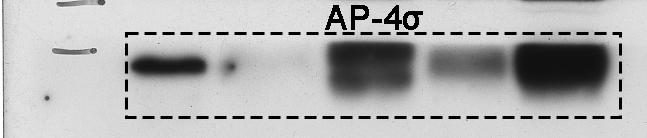

Supplement: Figure 2—source data 2. [file elife-76425-fig2-data2.zip › Figure 2 - source data 2/Figure 2B - AP4 sigma blot with cropped area.jpg]

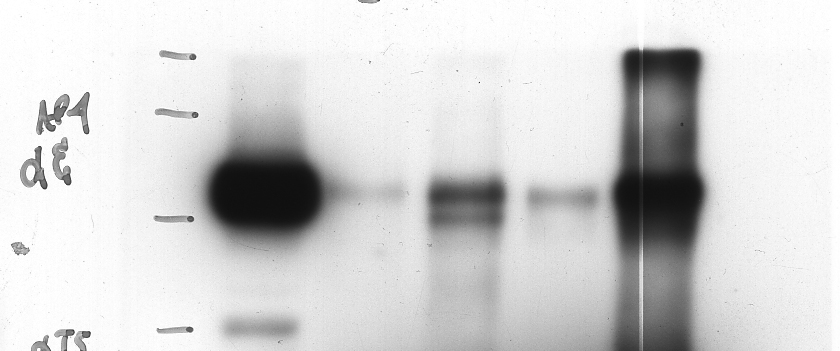

Supplement: Figure 2—source data 2. [file elife-76425-fig2-data2.zip › Figure 2 - source data 2/Figure 2B - AP4E blot raw image.jpg]

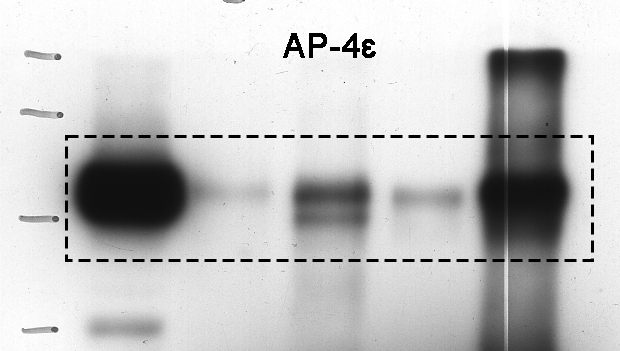

Supplement: Figure 2—source data 2. [file elife-76425-fig2-data2.zip › Figure 2 - source data 2/Figure 2B - AP4E blot with cropped area.jpg]

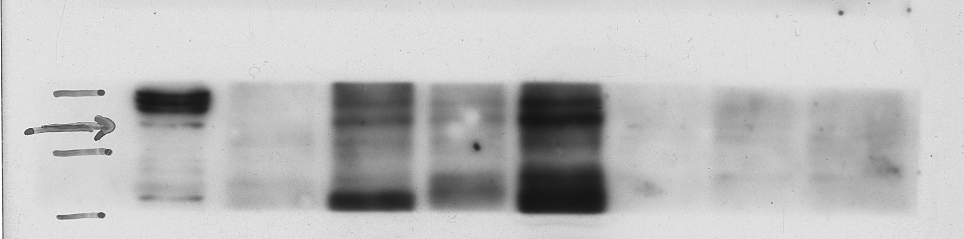

Supplement: Figure 2—source data 2. [file elife-76425-fig2-data2.zip › Figure 2 - source data 2/Figure 2B - TSPAN5 blot raw image.jpg]

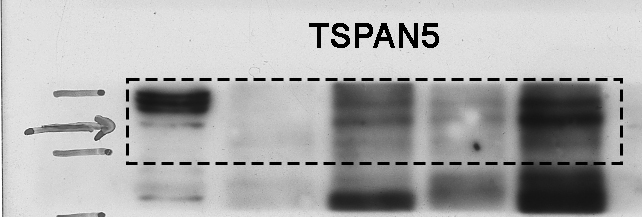

Supplement: Figure 2—source data 2. [file elife-76425-fig2-data2.zip › Figure 2 - source data 2/Figure 2B - TSPAN5 blot with cropped area.jpg]

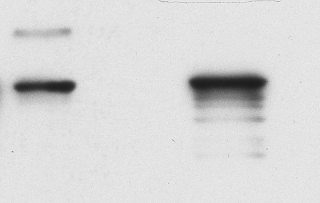

Supplement: Figure 2—source data 2. [file elife-76425-fig2-data2.zip › Figure 2 - source data 2/Figure 2C - GluA1 blot raw image.jpg]

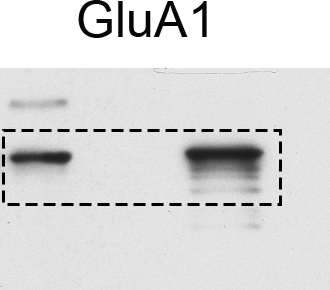

Supplement: Figure 2—source data 2. [file elife-76425-fig2-data2.zip › Figure 2 - source data 2/Figure 2C - GluA1 blot with cropped area.jpg]

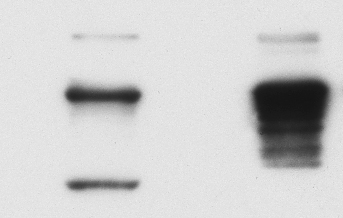

Supplement: Figure 2—source data 2. [file elife-76425-fig2-data2.zip › Figure 2 - source data 2/Figure 2C - GluA23 blot raw image.jpg]

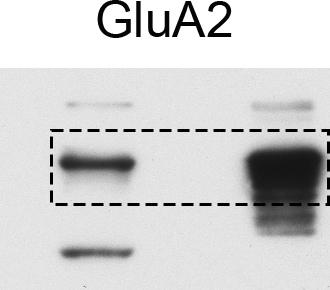

Supplement: Figure 2—source data 2. [file elife-76425-fig2-data2.zip › Figure 2 - source data 2/Figure 2C - GluA23 blot with cropped area.jpg]

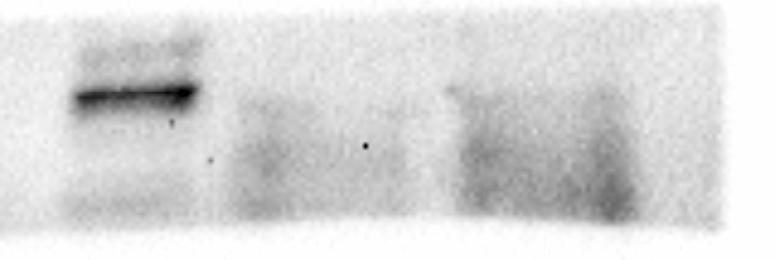

Supplement: Figure 2—source data 2. [file elife-76425-fig2-data2.zip › Figure 2 - source data 2/Figure 2C - GluN2A blot raw image.jpg]

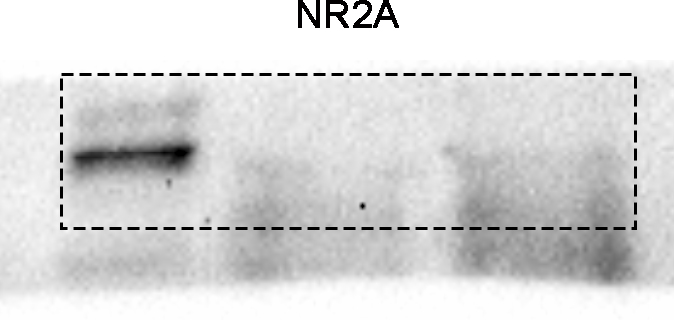

Supplement: Figure 2—source data 2. [file elife-76425-fig2-data2.zip › Figure 2 - source data 2/Figure 2C - GluN2A blot with cropped area.jpg]

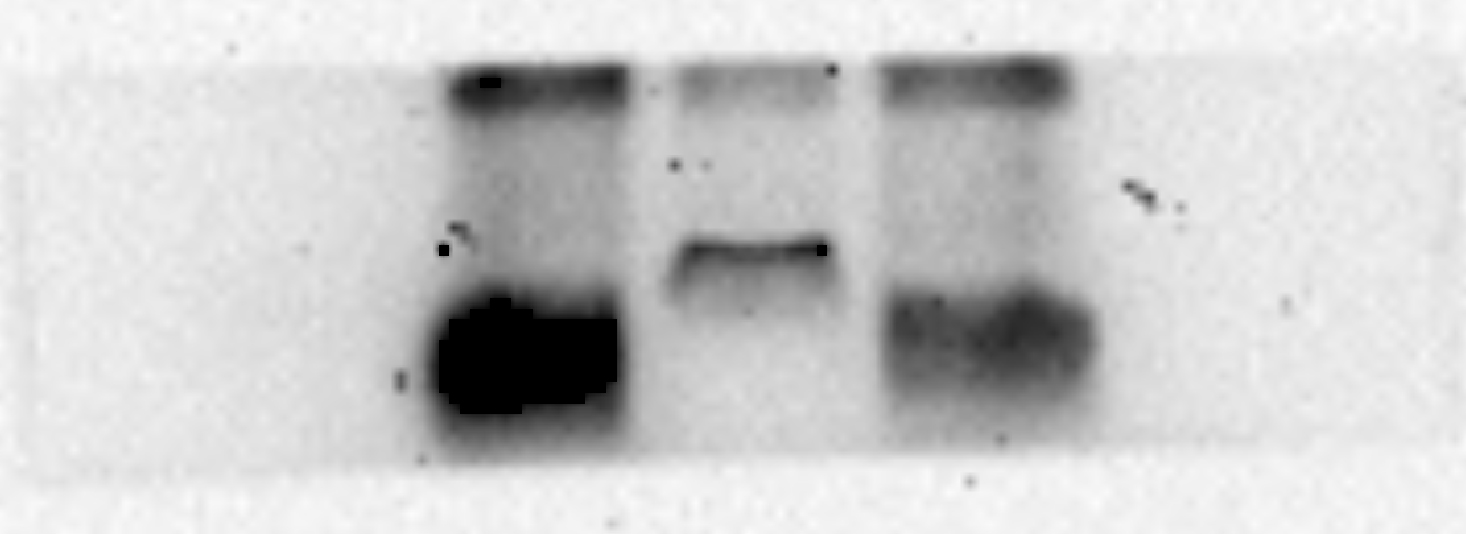

Supplement: Figure 2—source data 2. [file elife-76425-fig2-data2.zip › Figure 2 - source data 2/Figure 2C - Stargazin blot raw image.jpg]

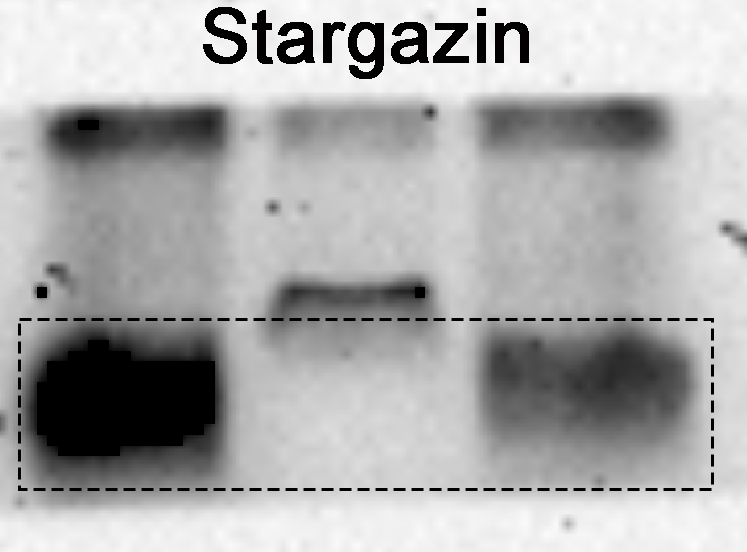

Supplement: Figure 2—source data 2. [file elife-76425-fig2-data2.zip › Figure 2 - source data 2/Figure 2C - Stargazin blot with cropped area.jpg]

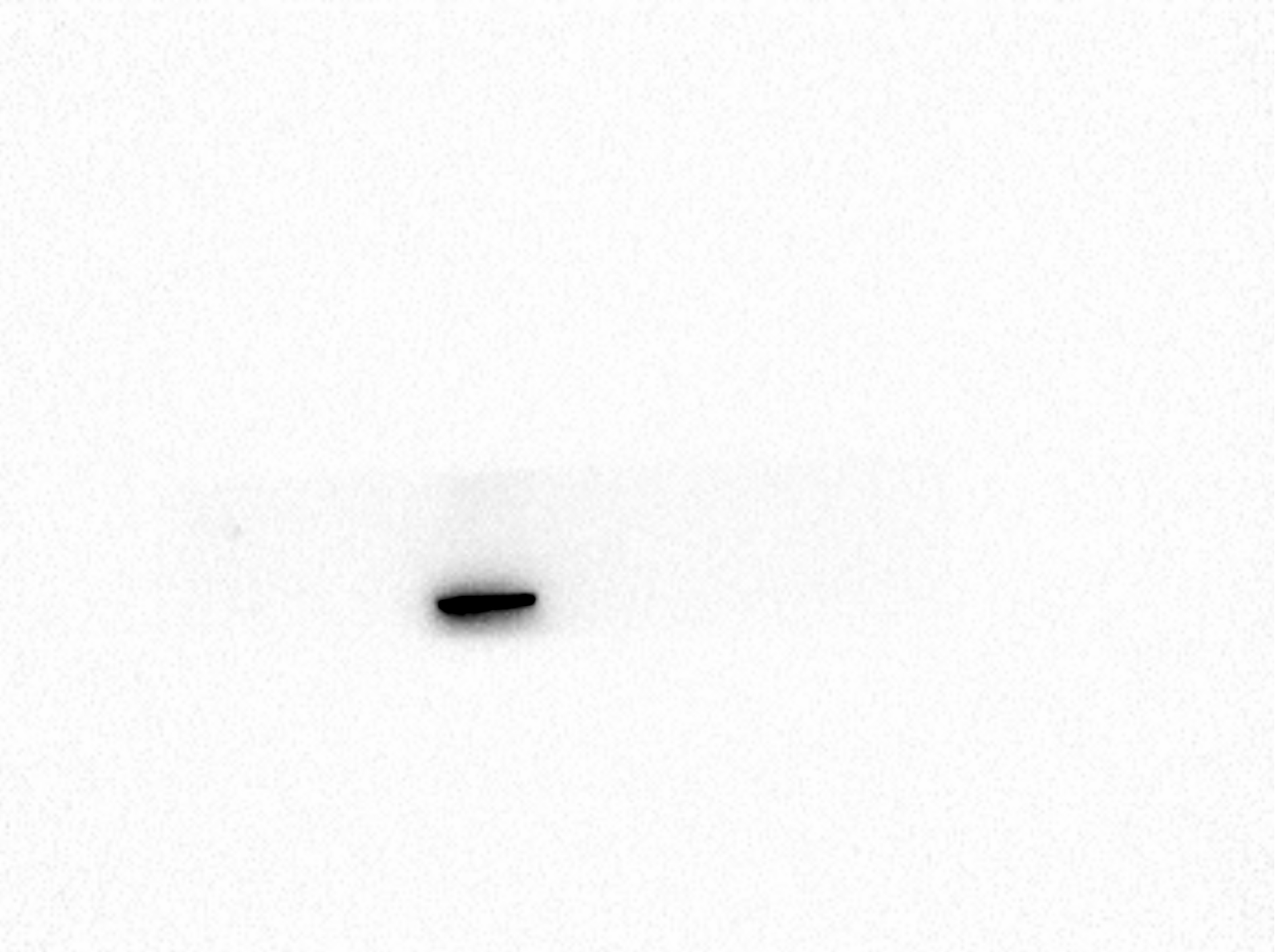

Supplement: Figure 2—source data 2. [file elife-76425-fig2-data2.zip › Figure 2 - source data 2/Figure 2E - CD81 blot raw image.jpg]

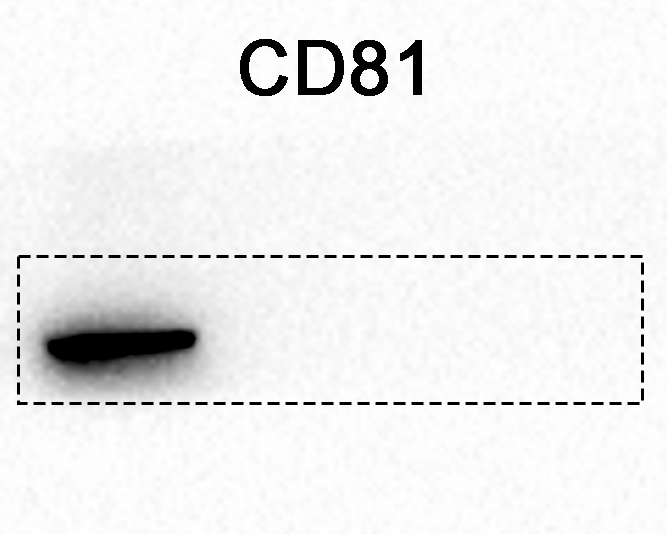

Supplement: Figure 2—source data 2. [file elife-76425-fig2-data2.zip › Figure 2 - source data 2/Figure 2E - CD81 blot with cropped area.jpg]

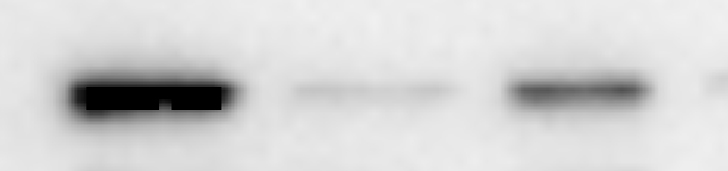

Supplement: Figure 2—source data 2. [file elife-76425-fig2-data2.zip › Figure 2 - source data 2/Figure 2E - GluA23 blot raw image.jpg]

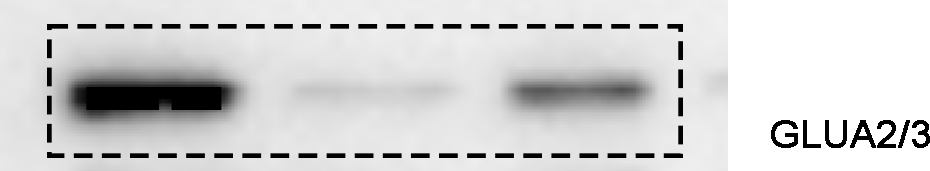

Supplement: Figure 2—source data 2. [file elife-76425-fig2-data2.zip › Figure 2 - source data 2/Figure 2E - GluA23 blot with cropped area.jpg]

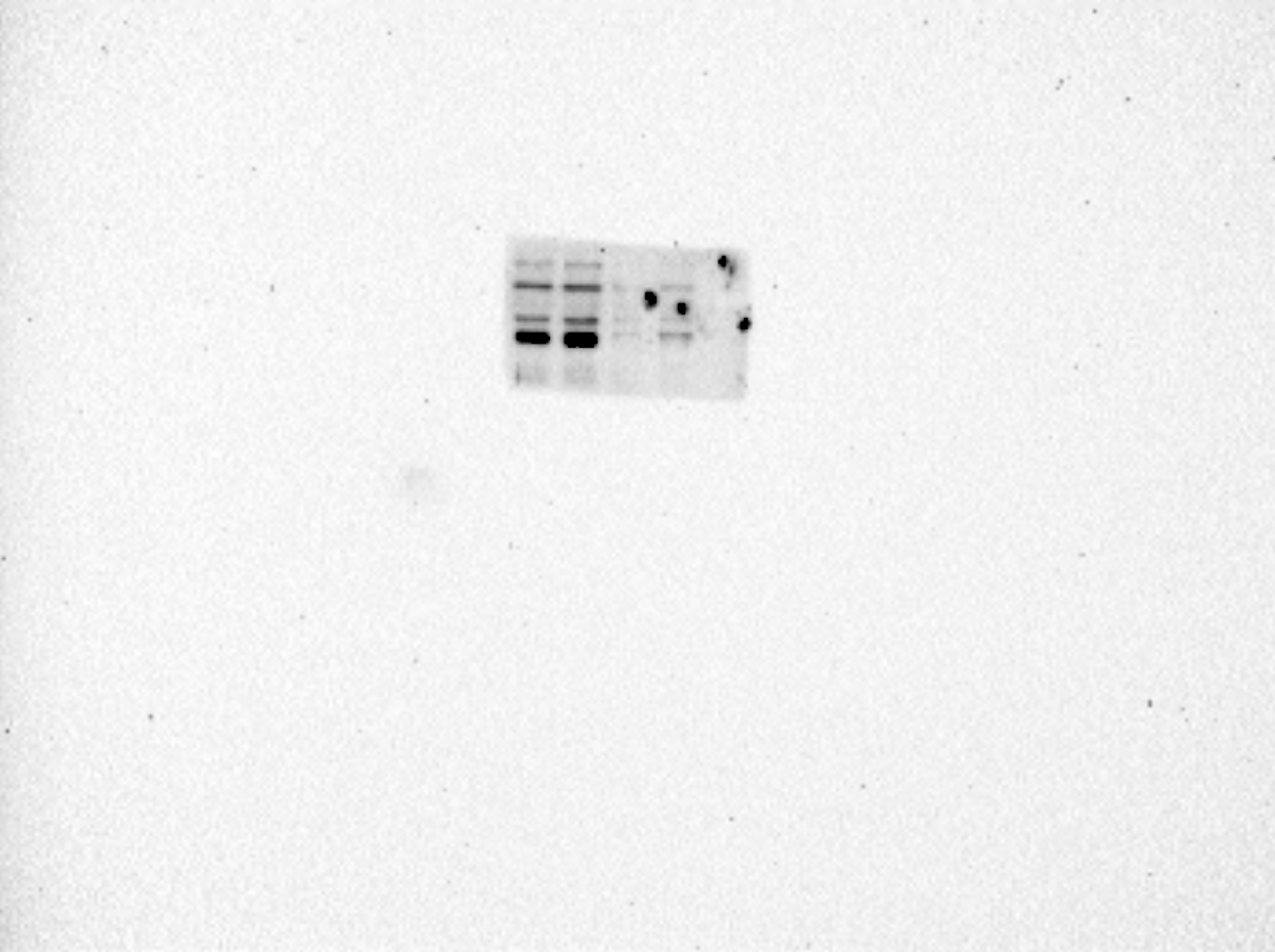

Supplement: Figure 2—source data 2. [file elife-76425-fig2-data2.zip › Figure 2 - source data 2/Figure 2E - TSPAN5 blot raw image.jpg]

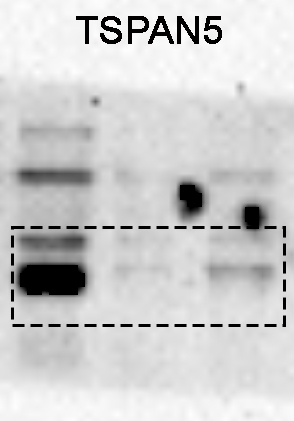

Supplement: Figure 2—source data 2. [file elife-76425-fig2-data2.zip › Figure 2 - source data 2/Figure 2E - TSPAN5 blot with cropped area.jpg]

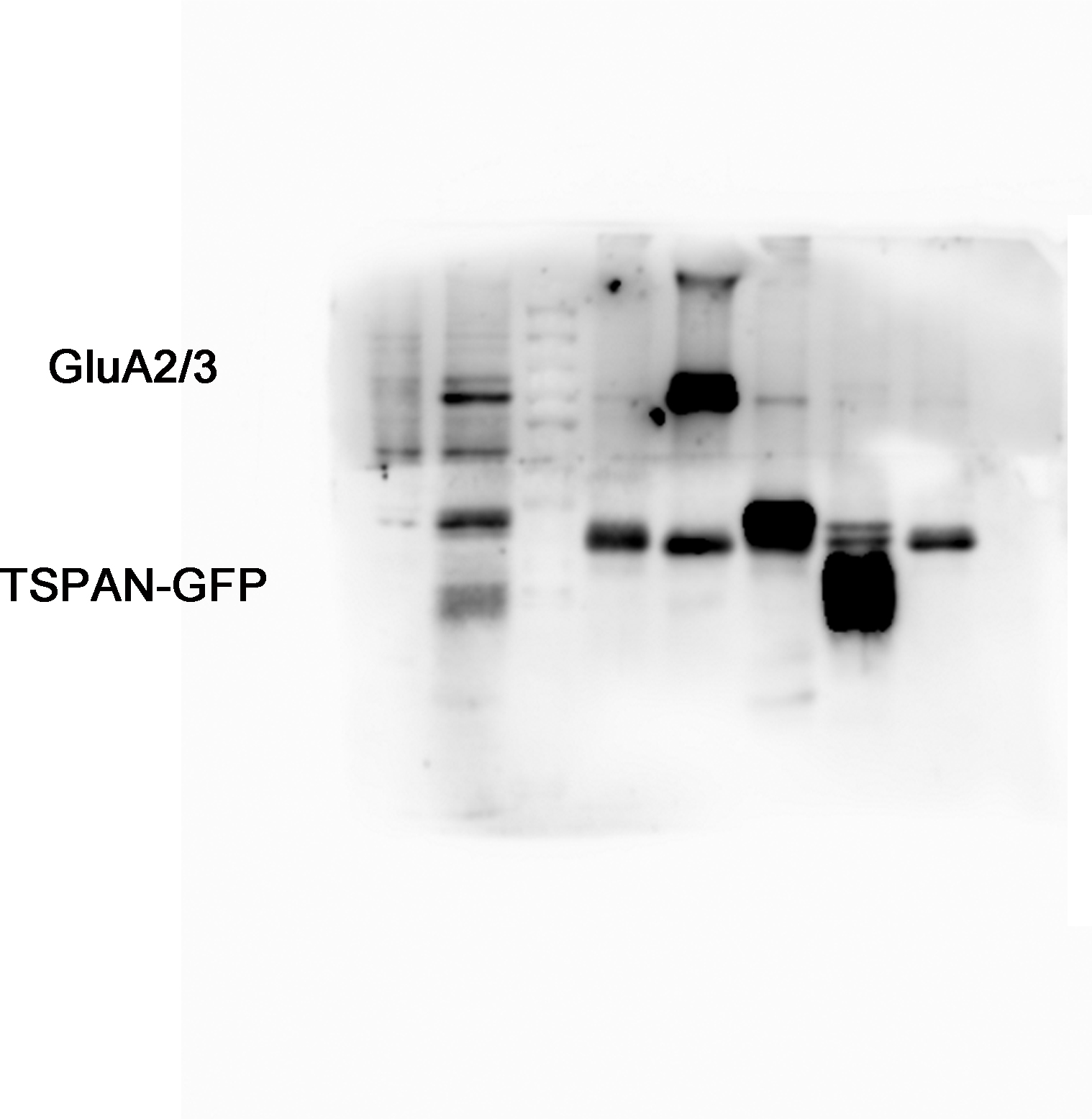

Supplement: Figure 2—figure supplement 1—source data 1. [file elife-76425-fig2-figsupp1-data1.zip › Figure 2 - Figure Supplement 1 - source data/GluA23 and TSPAN5-GFP blots raw data.jpg]

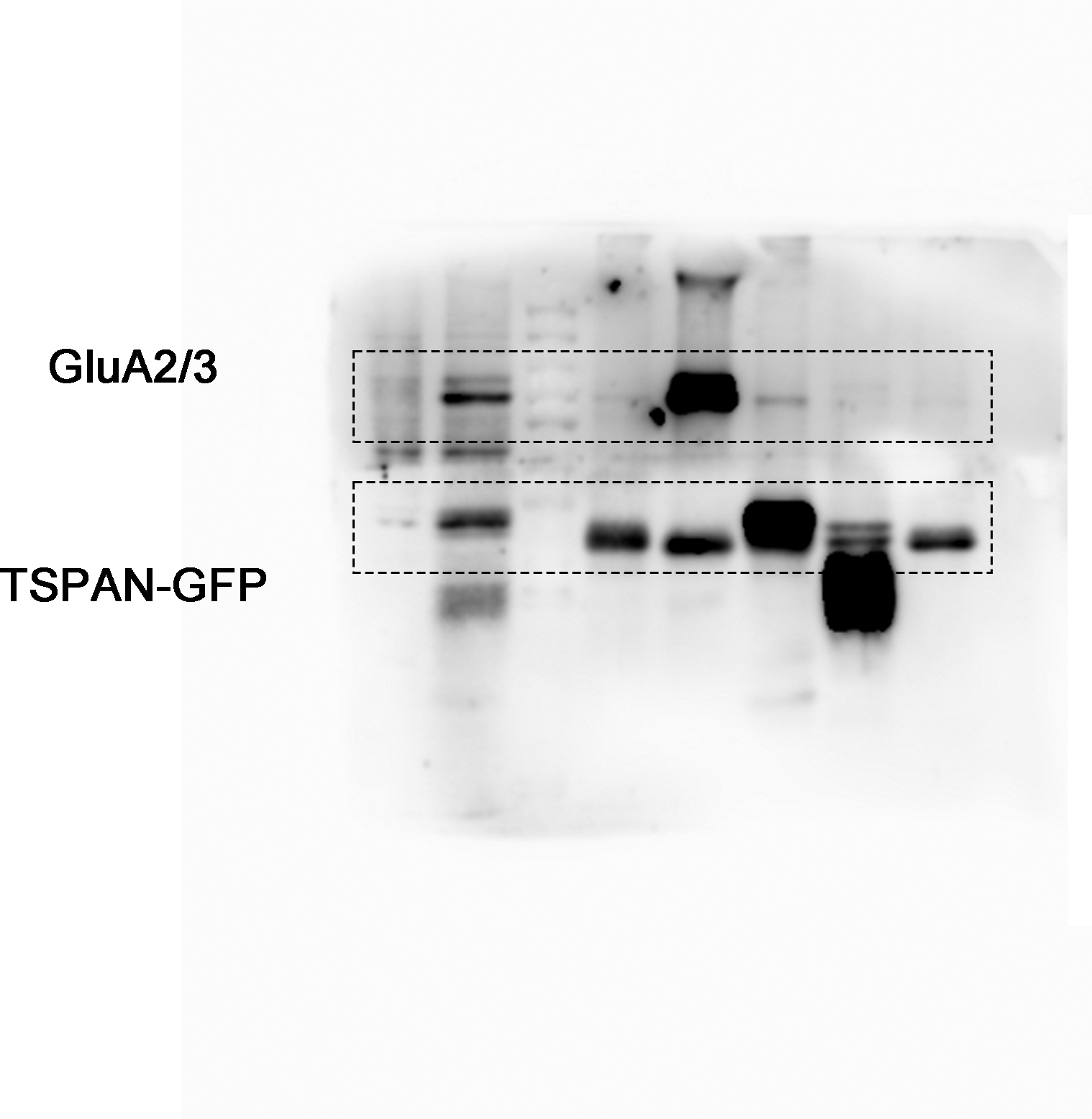

Supplement: Figure 2—figure supplement 1—source data 1. [file elife-76425-fig2-figsupp1-data1.zip › Figure 2 - Figure Supplement 1 - source data/GluA23 and TSPAN5-GFP blots with cropped area.jpg]

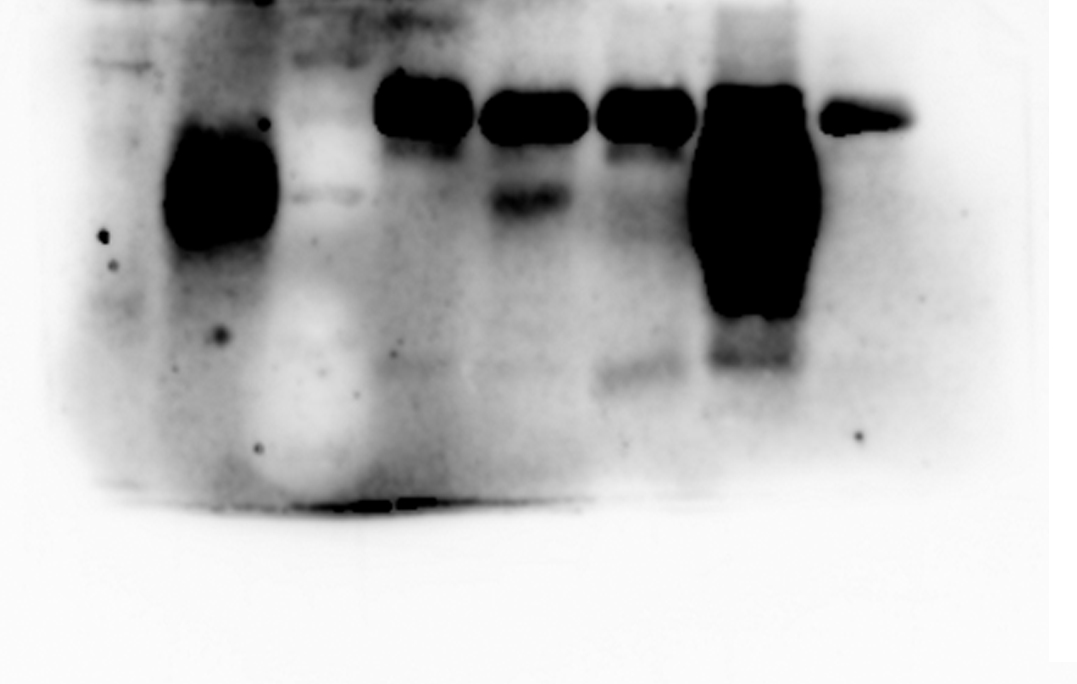

Supplement: Figure 2—figure supplement 1—source data 1. [file elife-76425-fig2-figsupp1-data1.zip › Figure 2 - Figure Supplement 1 - source data/Stargazin HA blot raw data.jpg]

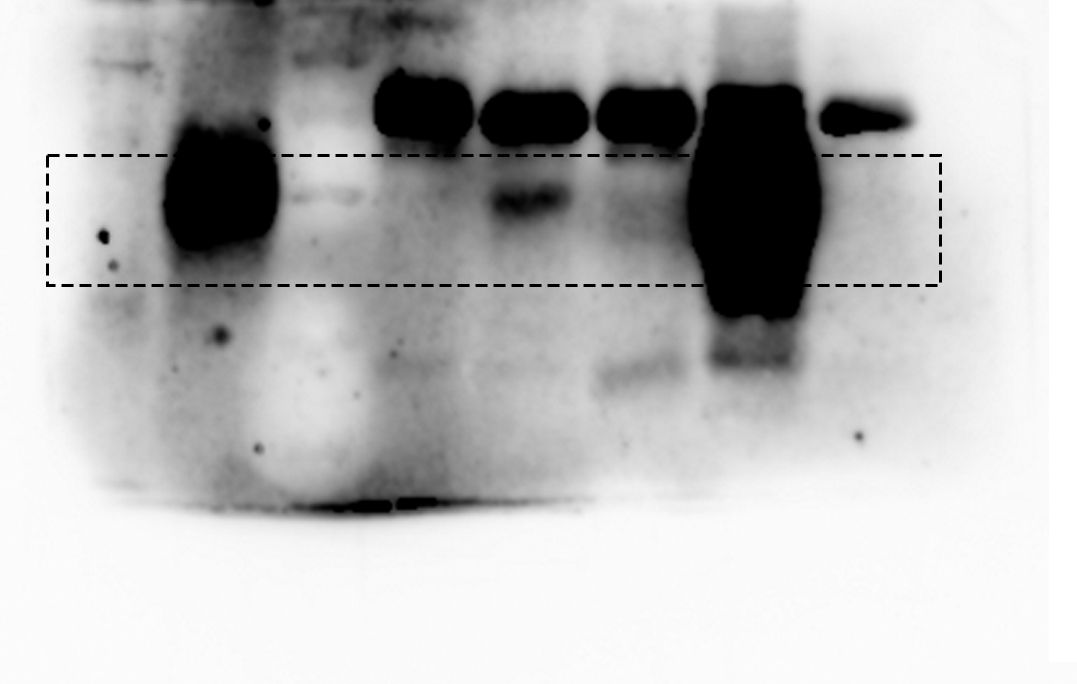

Supplement: Figure 2—figure supplement 1—source data 1. [file elife-76425-fig2-figsupp1-data1.zip › Figure 2 - Figure Supplement 1 - source data/Stargazin HA blot with cropped area.jpg]

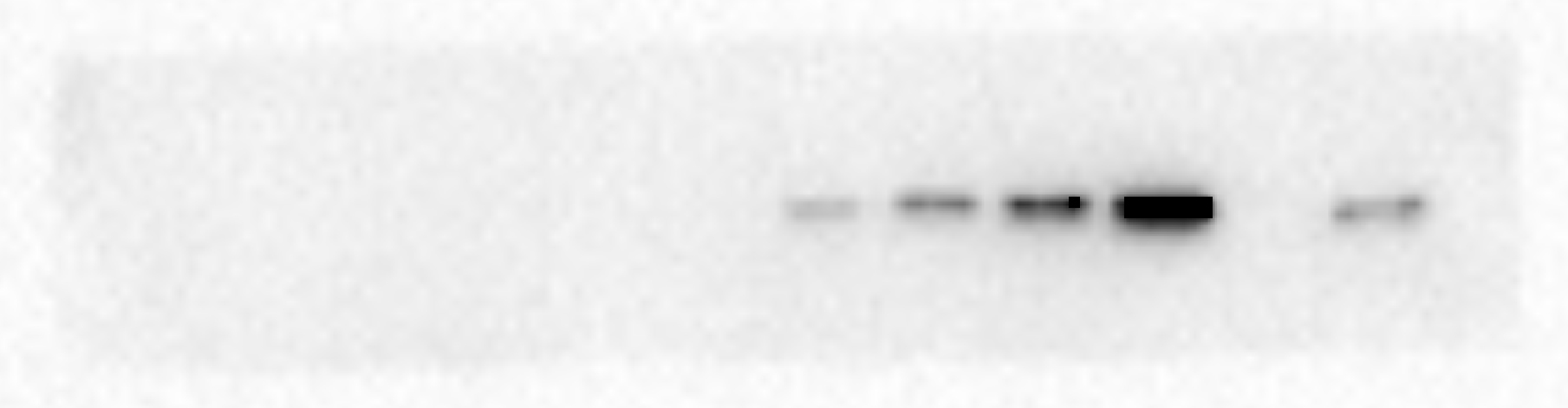

Supplement: Figure 3—source data 2. [file elife-76425-fig3-data2.zip › Figure 3 - source data 2/Figure 3A - AP4E blot raw image.jpg]

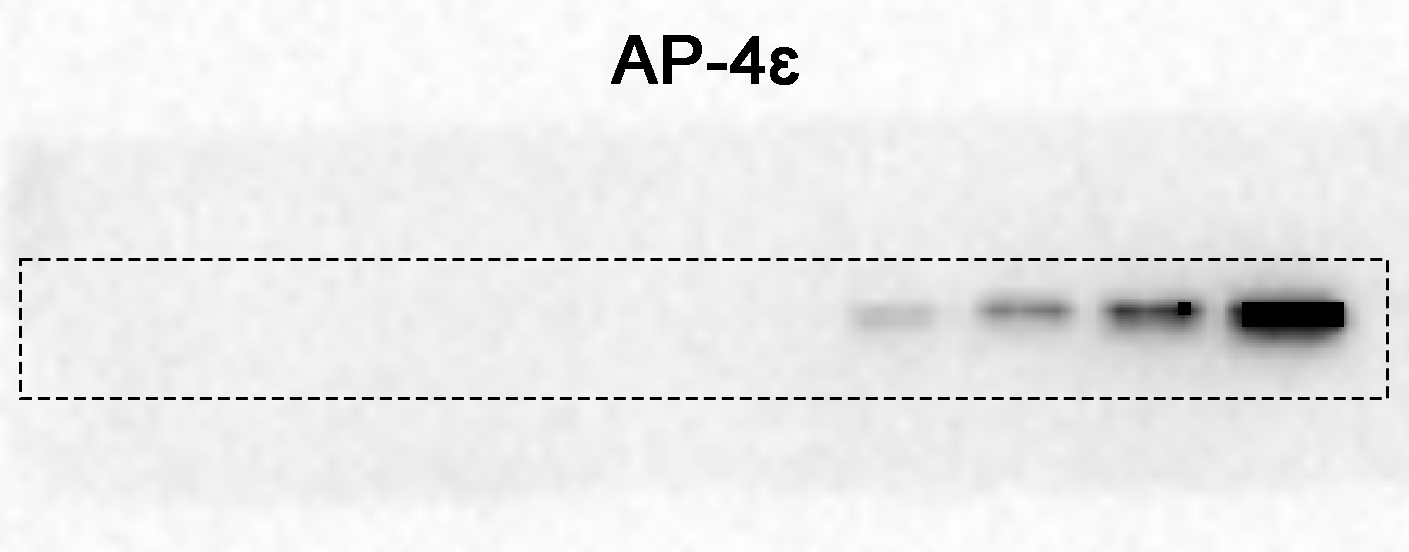

Supplement: Figure 3—source data 2. [file elife-76425-fig3-data2.zip › Figure 3 - source data 2/Figure 3A - AP4E blot with cropped area.jpg]

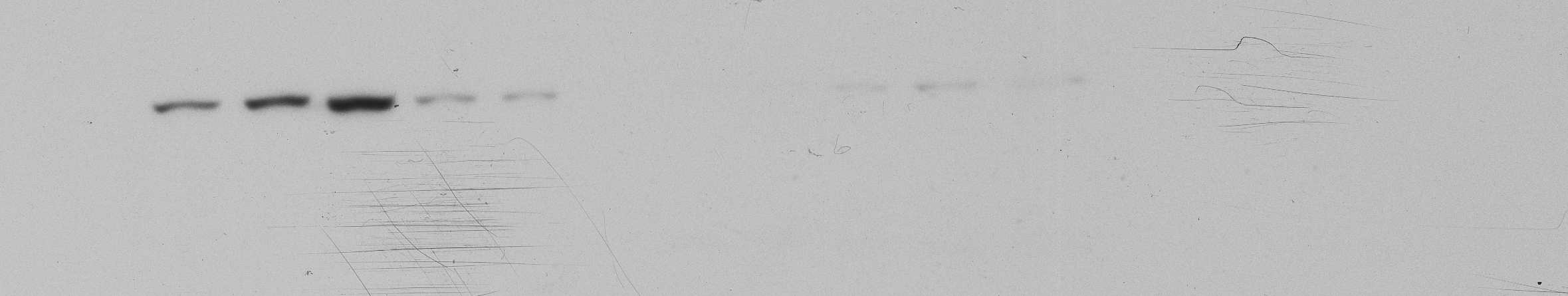

Supplement: Figure 3—source data 2. [file elife-76425-fig3-data2.zip › Figure 3 - source data 2/Figure 3A - EEA1 blot raw image.jpg]

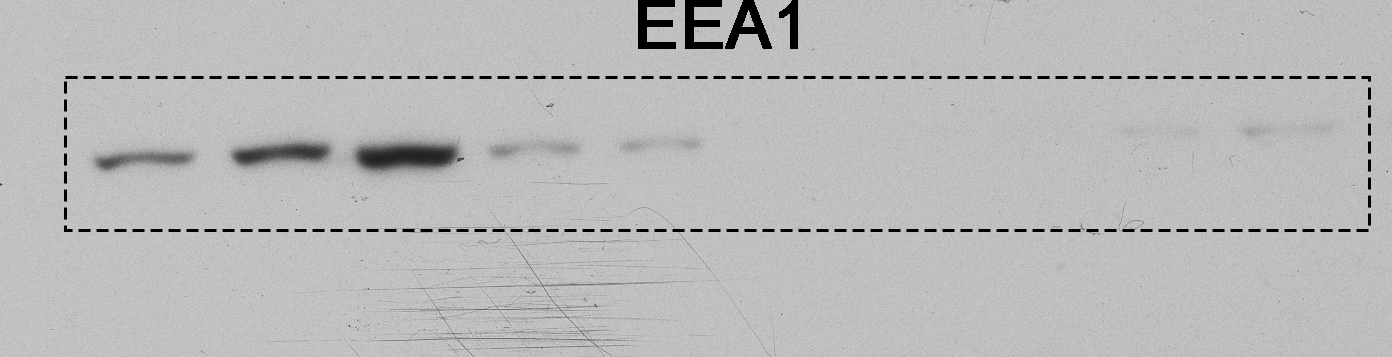

Supplement: Figure 3—source data 2. [file elife-76425-fig3-data2.zip › Figure 3 - source data 2/Figure 3A - EEA1 blot with cropped area.jpg]

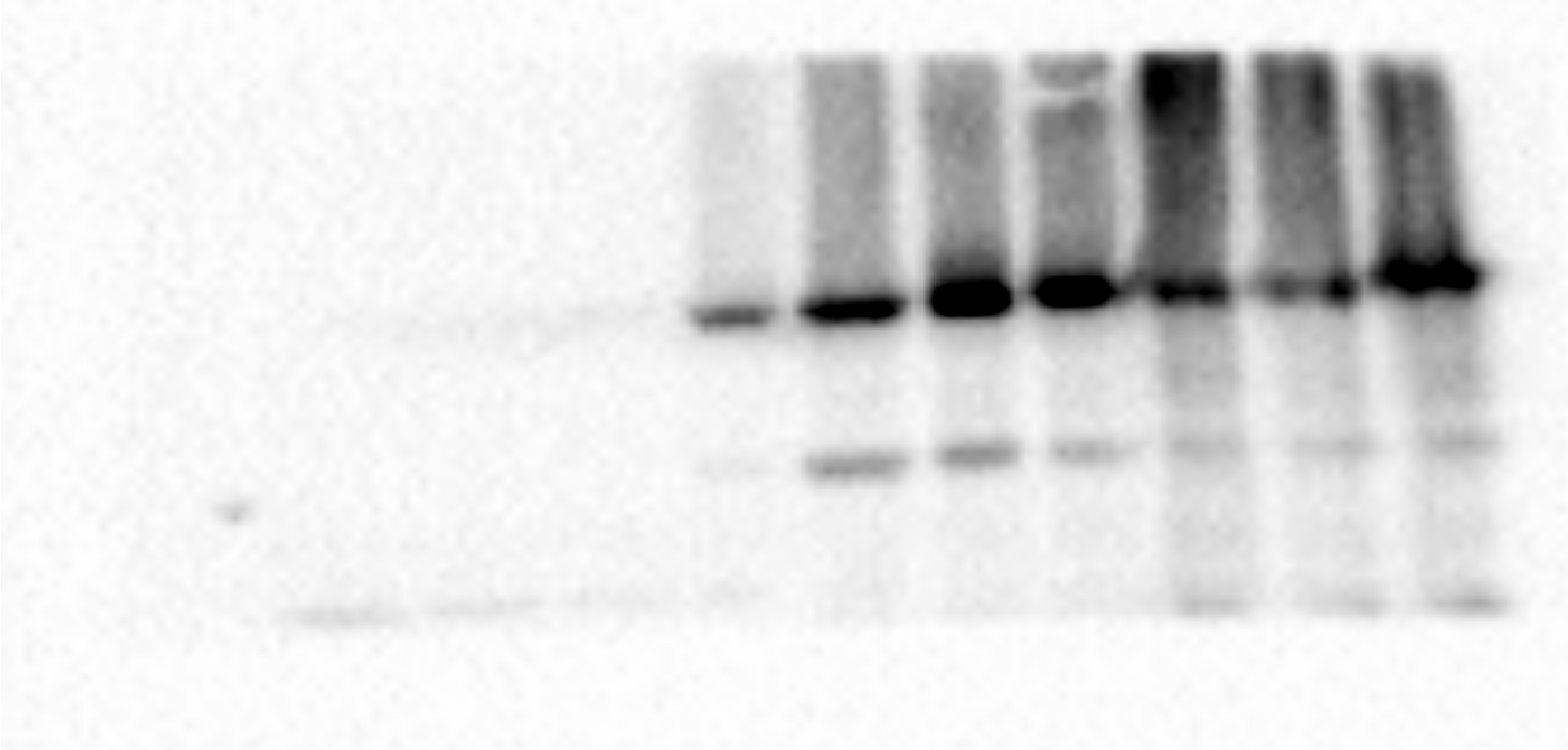

Supplement: Figure 3—source data 2. [file elife-76425-fig3-data2.zip › Figure 3 - source data 2/Figure 3A - GluA1 blot raw image.jpg]

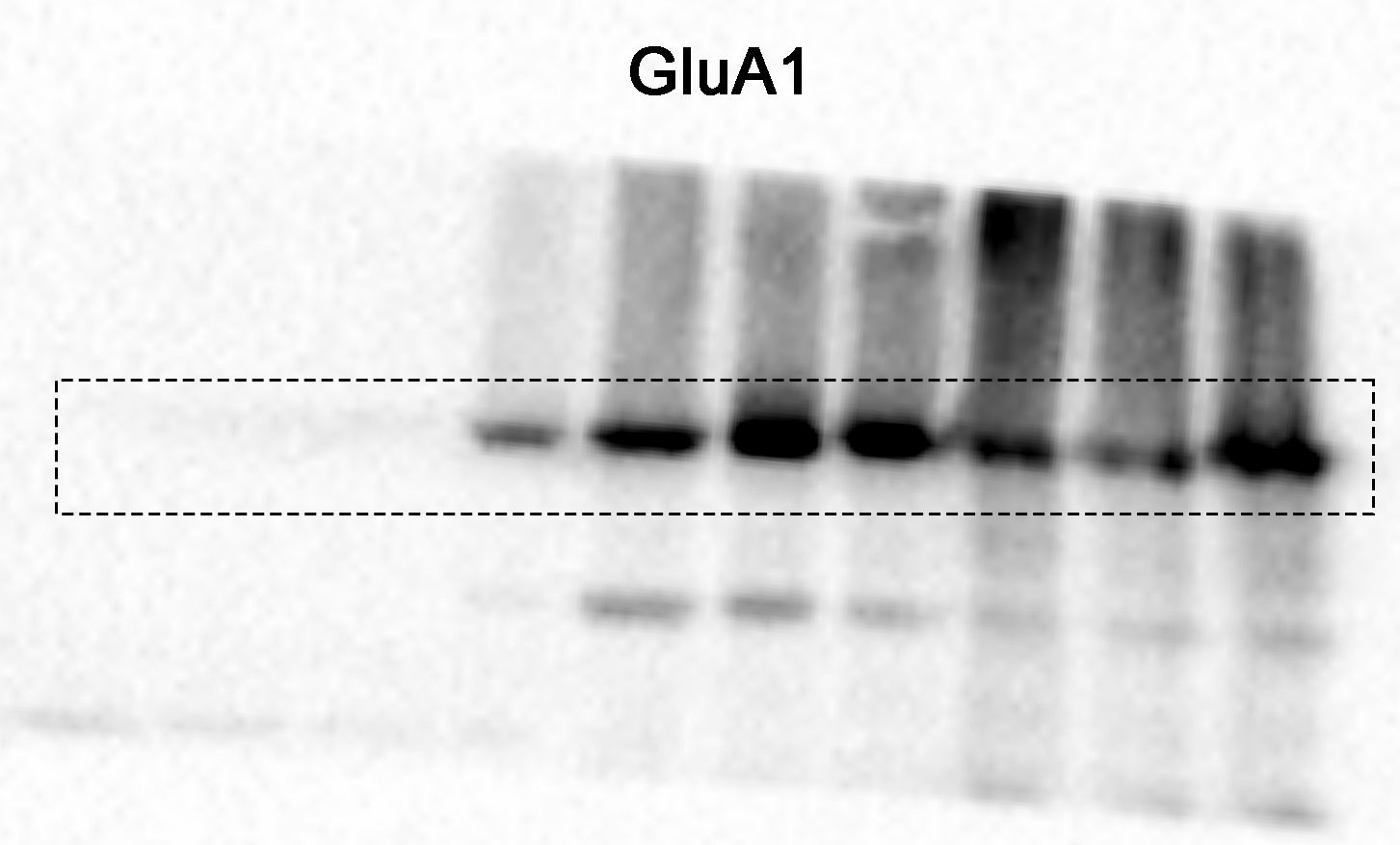

Supplement: Figure 3—source data 2. [file elife-76425-fig3-data2.zip › Figure 3 - source data 2/Figure 3A - GluA1 blot with cropped area.jpg]

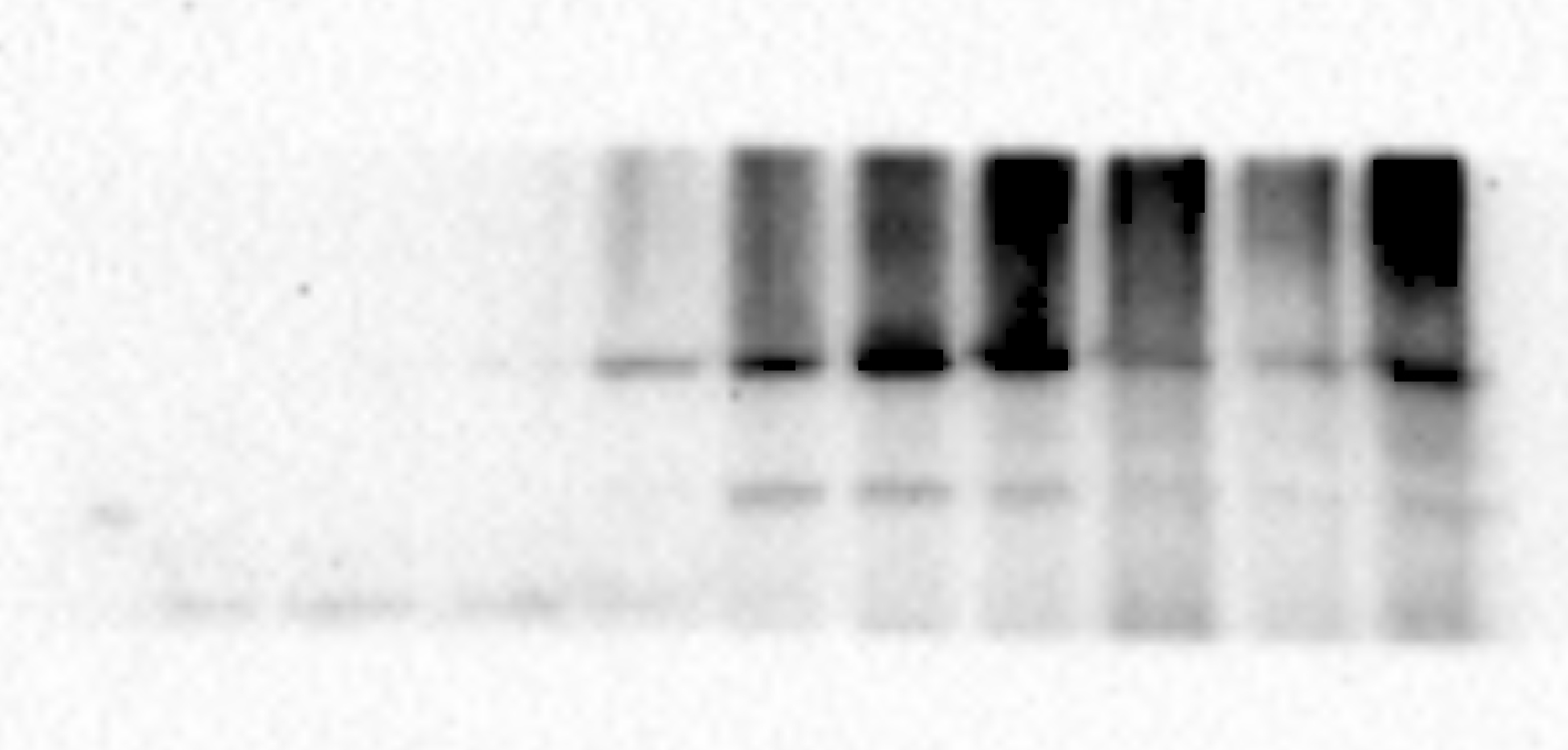

Supplement: Figure 3—source data 2. [file elife-76425-fig3-data2.zip › Figure 3 - source data 2/Figure 3A - GluA23 blot raw image.jpg]

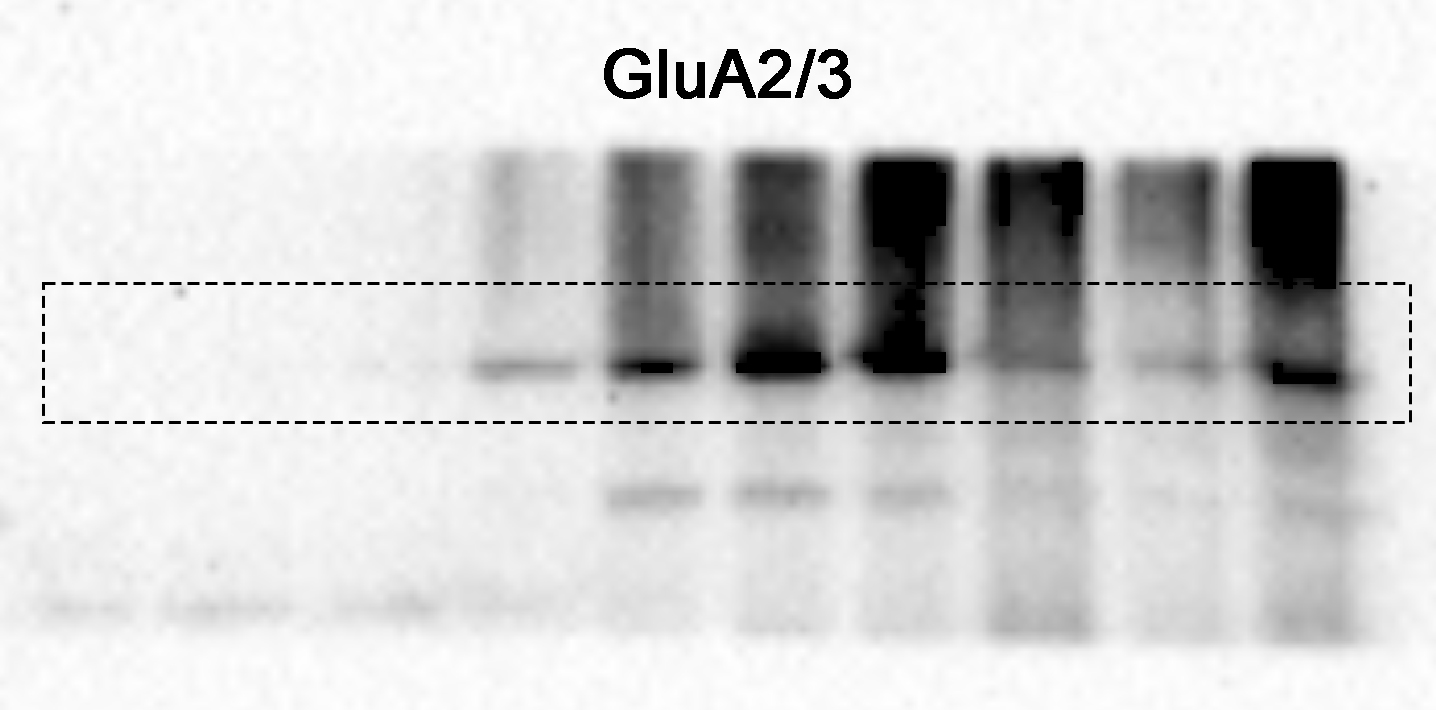

Supplement: Figure 3—source data 2. [file elife-76425-fig3-data2.zip › Figure 3 - source data 2/Figure 3A - GluA23 blot with cropped area.jpg]

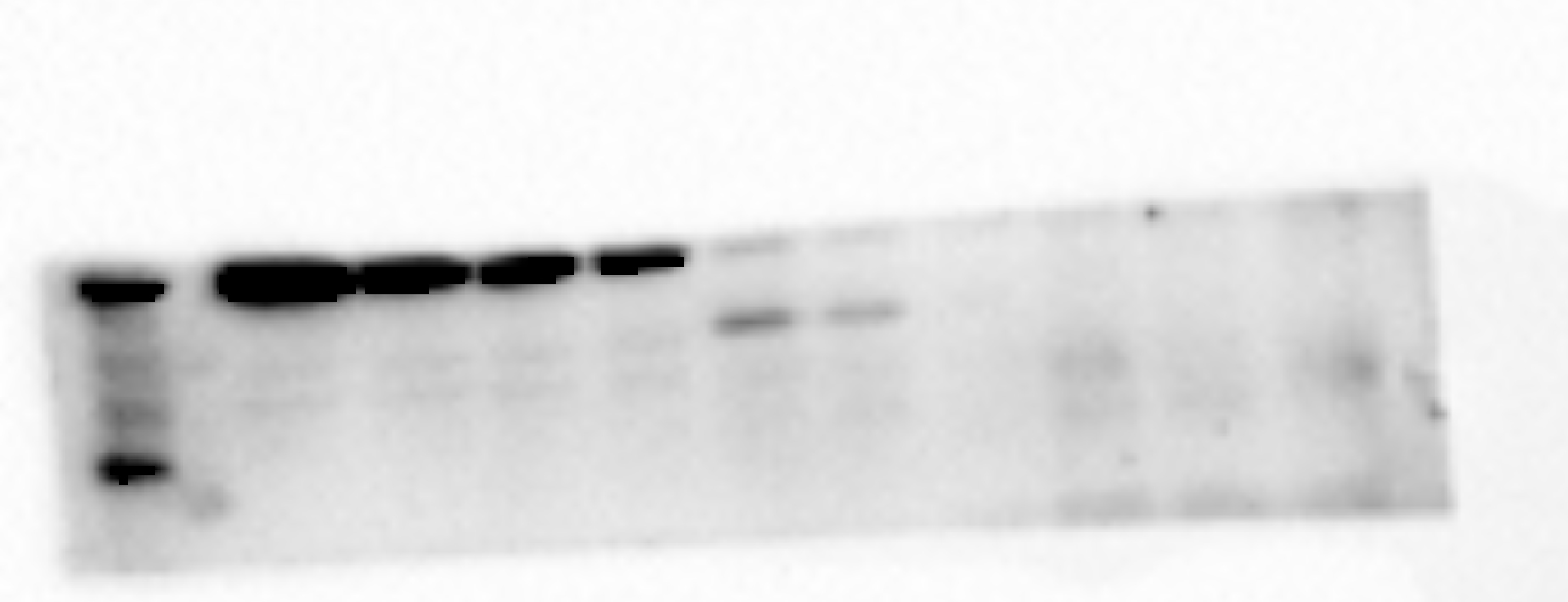

Supplement: Figure 3—source data 2. [file elife-76425-fig3-data2.zip › Figure 3 - source data 2/Figure 3A - Rab7 blot raw image.jpg]

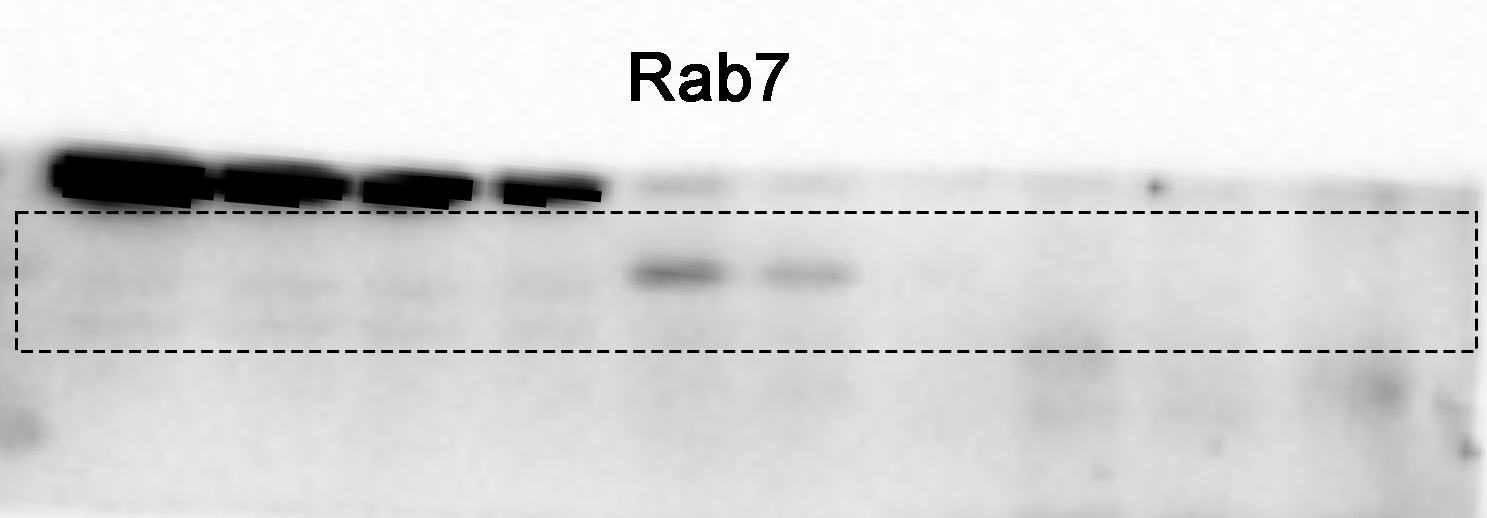

Supplement: Figure 3—source data 2. [file elife-76425-fig3-data2.zip › Figure 3 - source data 2/Figure 3A - Rab7 blot with cropped area.jpg]

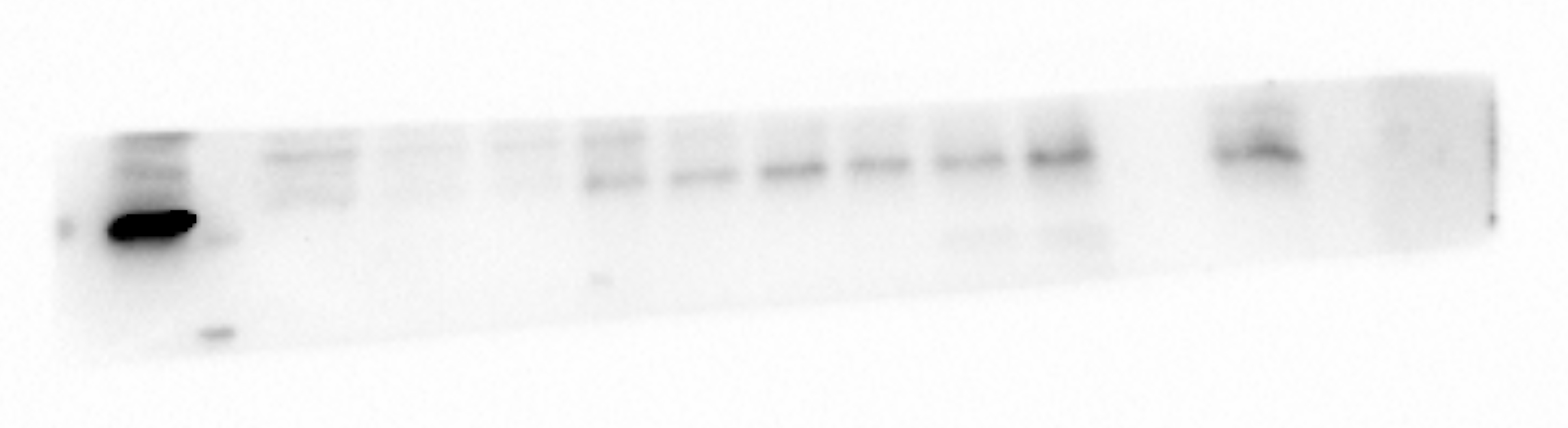

Supplement: Figure 3—source data 2. [file elife-76425-fig3-data2.zip › Figure 3 - source data 2/Figure 3A - Stargazin blot raw image.jpg]

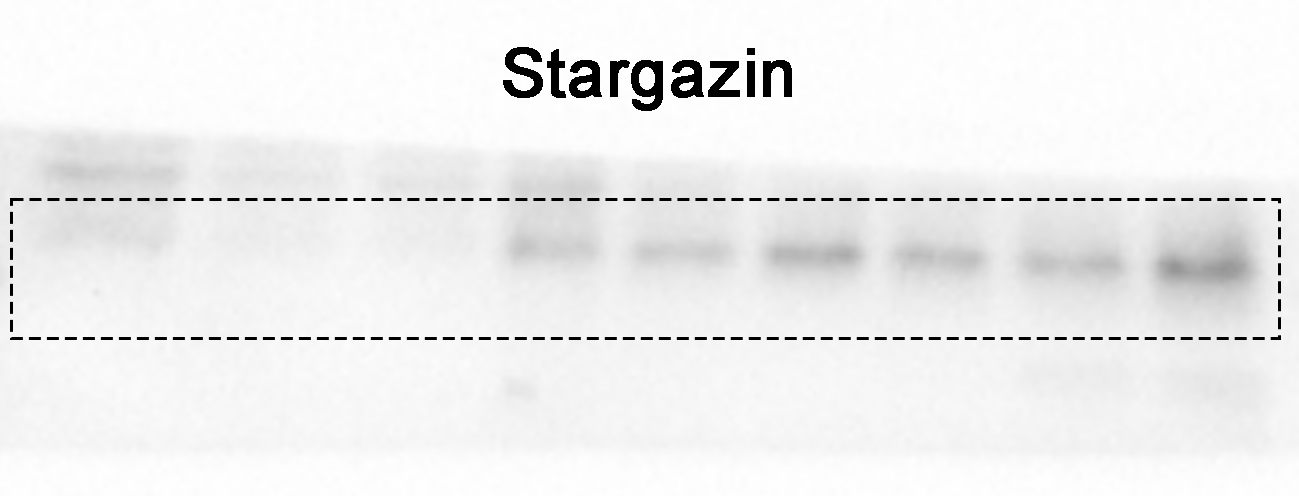

Supplement: Figure 3—source data 2. [file elife-76425-fig3-data2.zip › Figure 3 - source data 2/Figure 3A - Stargazin blot with cropped area.jpg]

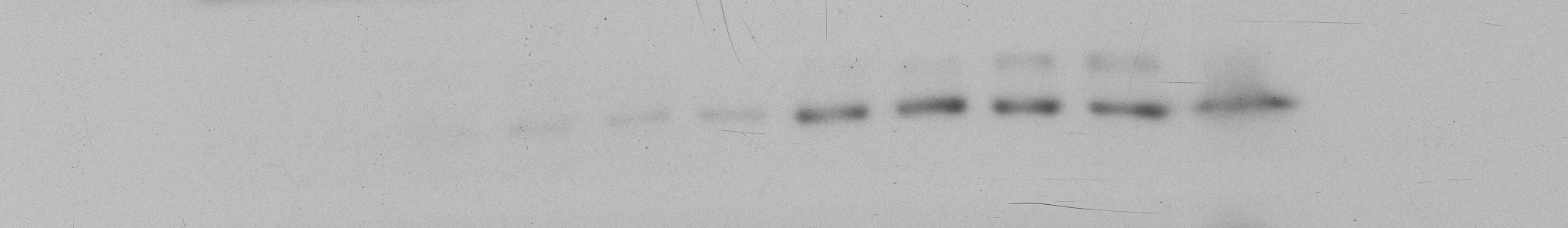

Supplement: Figure 3—source data 2. [file elife-76425-fig3-data2.zip › Figure 3 - source data 2/Figure 3A - TfR blot raw image.jpg]

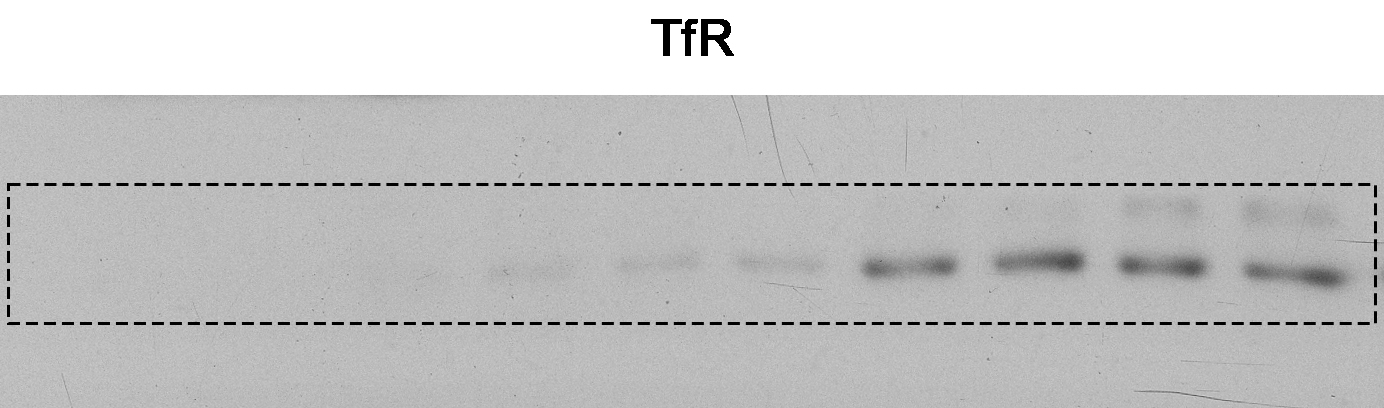

Supplement: Figure 3—source data 2. [file elife-76425-fig3-data2.zip › Figure 3 - source data 2/Figure 3A - TfR blot with cropped area.jpg]

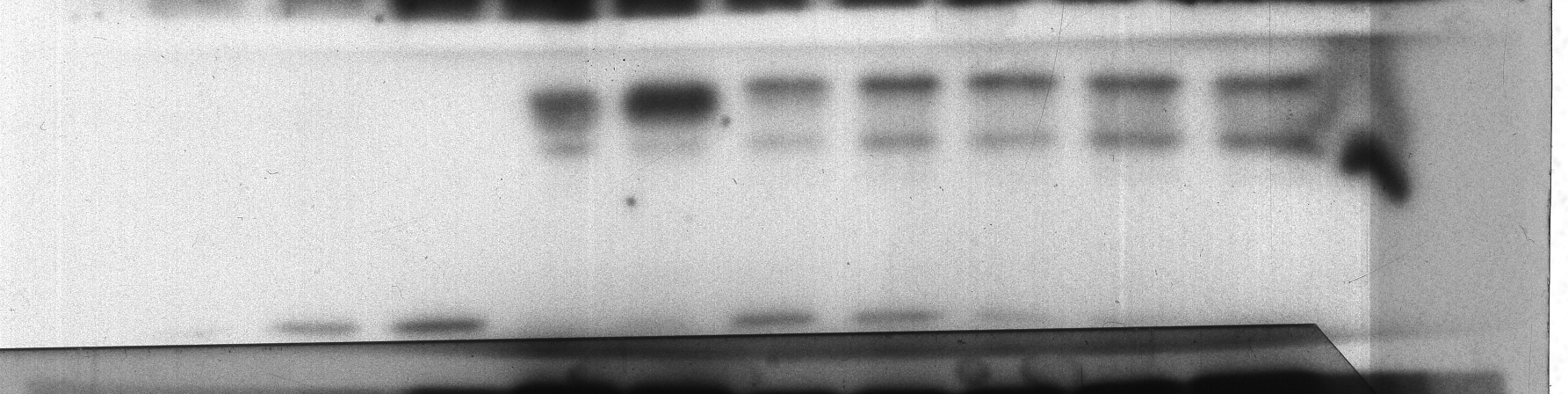

Supplement: Figure 3—source data 2. [file elife-76425-fig3-data2.zip › Figure 3 - source data 2/Figure 3A - TSPAN5 blot raw image.jpg]

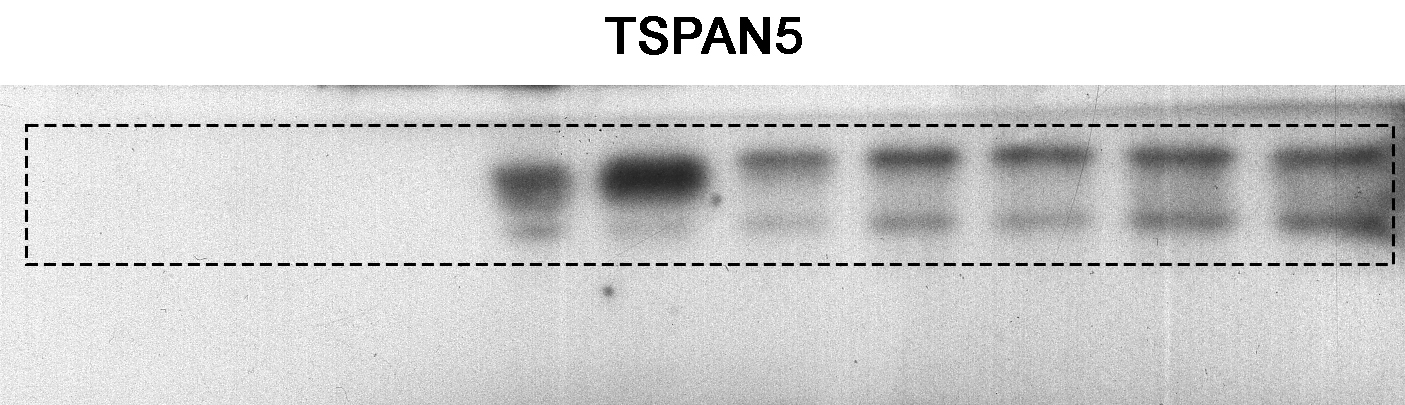

Supplement: Figure 3—source data 2. [file elife-76425-fig3-data2.zip › Figure 3 - source data 2/Figure 3A - TSPAN5 blot with cropped area.jpg]

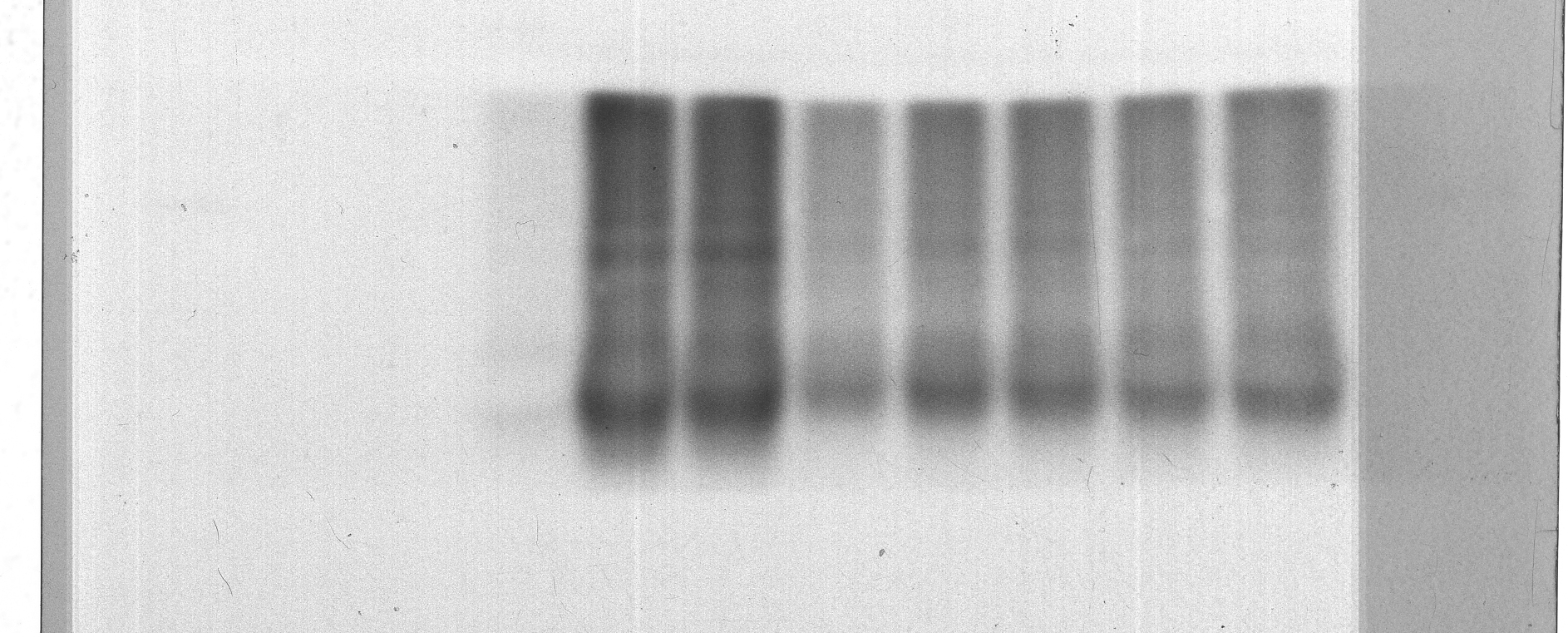

Supplement: Figure 3—source data 2. [file elife-76425-fig3-data2.zip › Figure 3 - source data 2/Figure 3A - VGlut1 blot raw image.jpg]

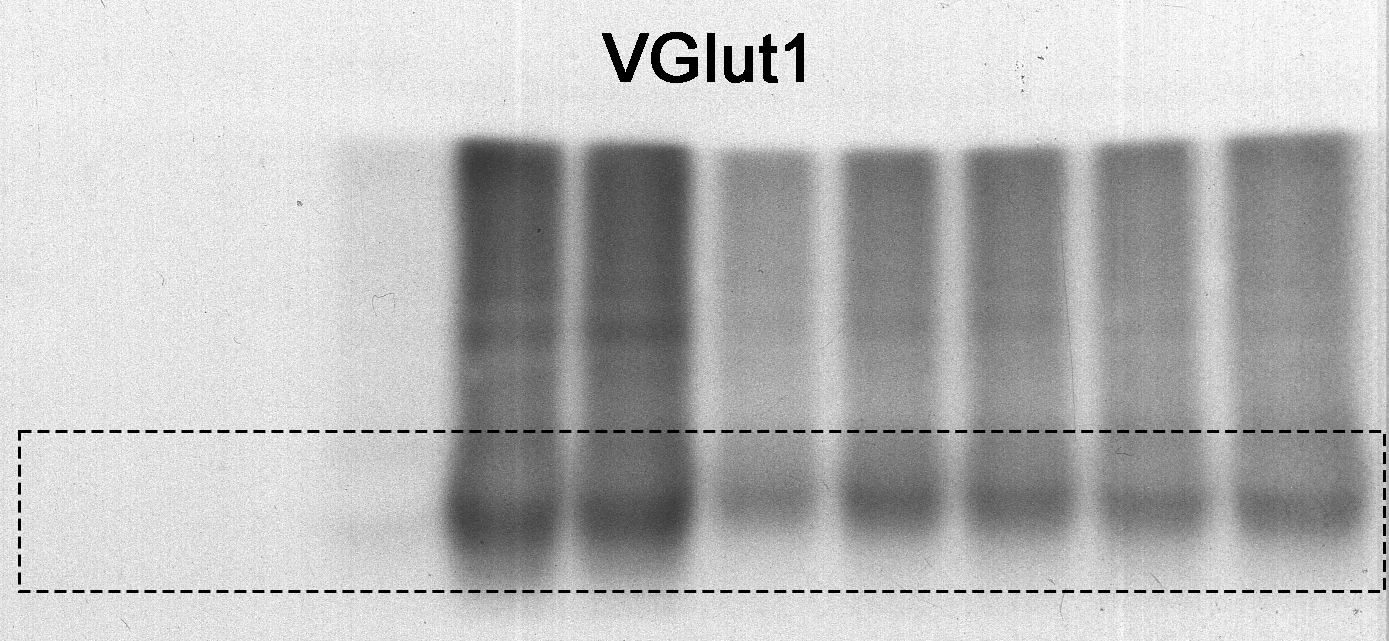

Supplement: Figure 3—source data 2. [file elife-76425-fig3-data2.zip › Figure 3 - source data 2/Figure 3A - VGlut1 blot with cropped area.jpg]

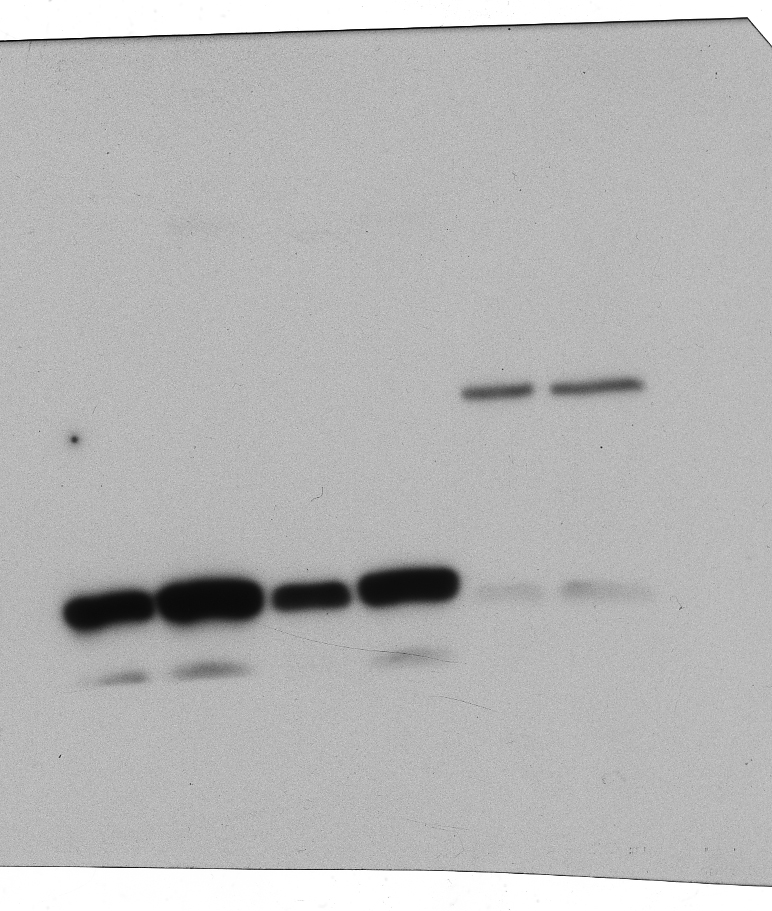

Supplement: Figure 4—source data 2. [file elife-76425-fig4-data2.zip › Figure 4 - source data 2/Figure 4C - GFP blot raw data.jpg]

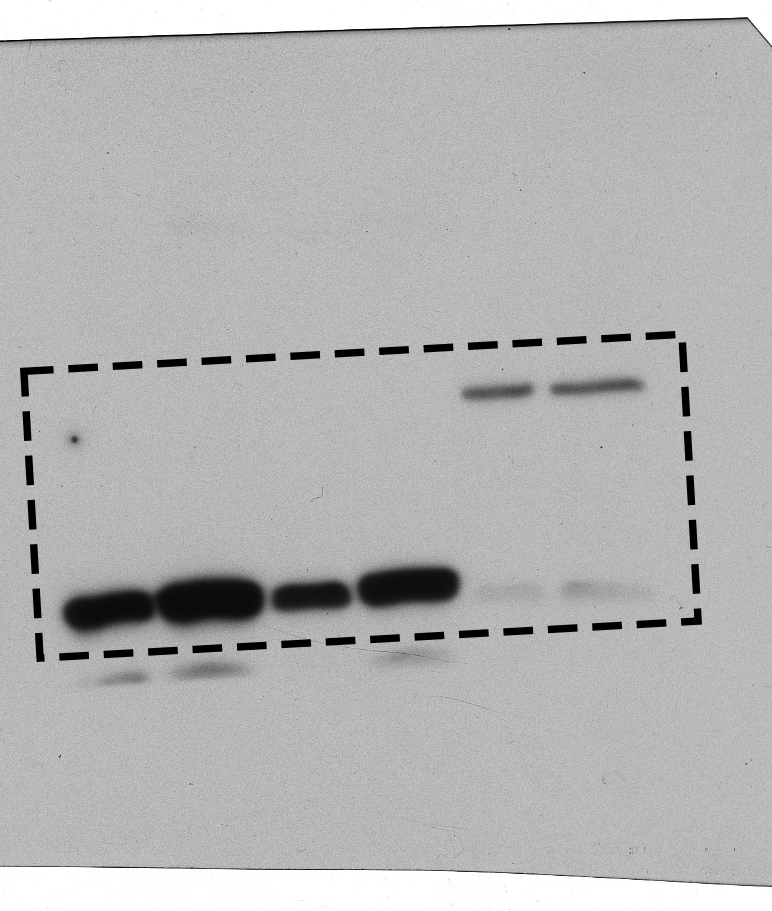

Supplement: Figure 4—source data 2. [file elife-76425-fig4-data2.zip › Figure 4 - source data 2/Figure 4C - GFP blot with cropped area copia.jpg]

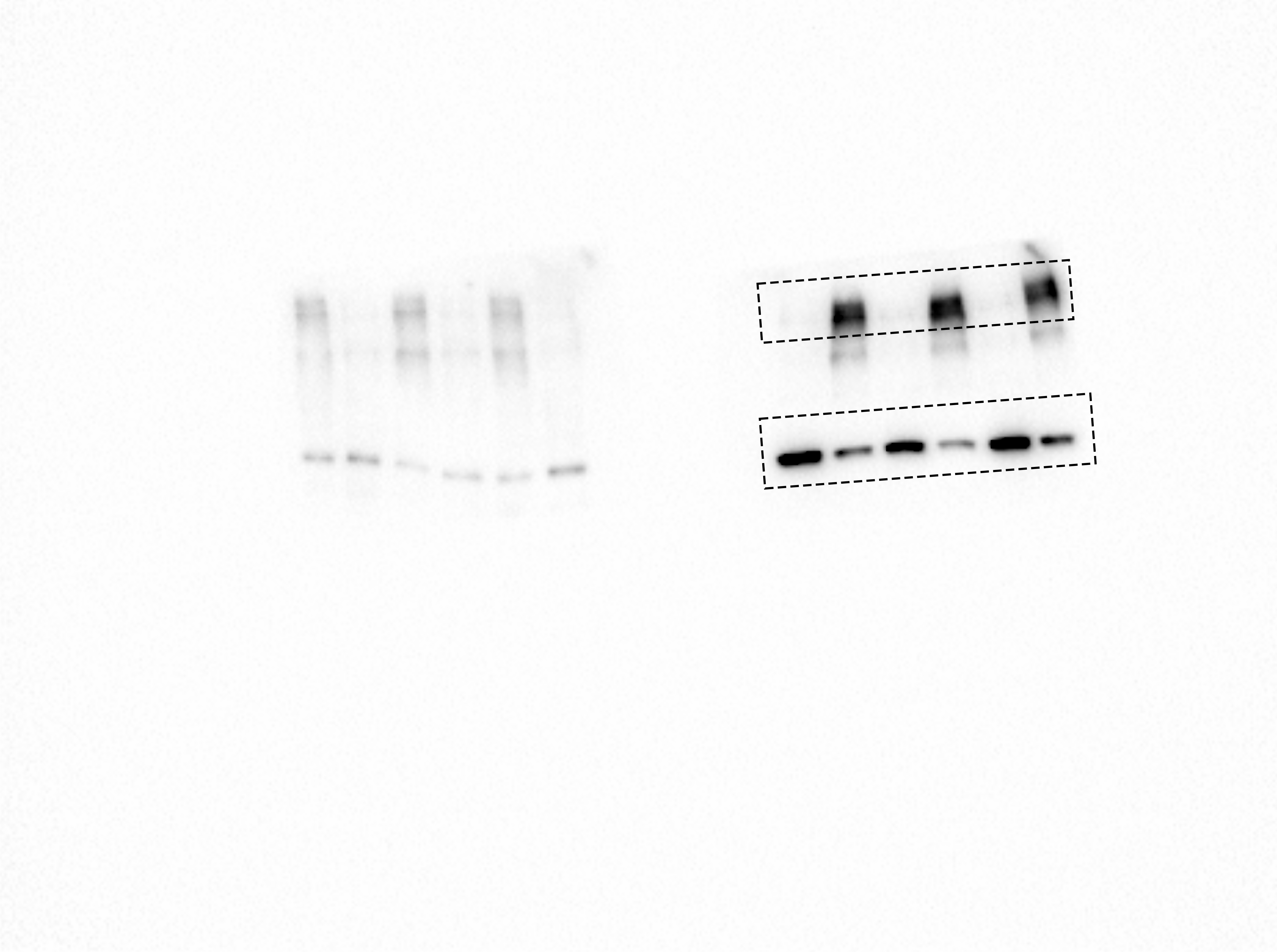

Supplement: Figure 4—source data 2. [file elife-76425-fig4-data2.zip › Figure 4 - source data 2/Figure 4C - GluA1 blot with cropped area.jpg]

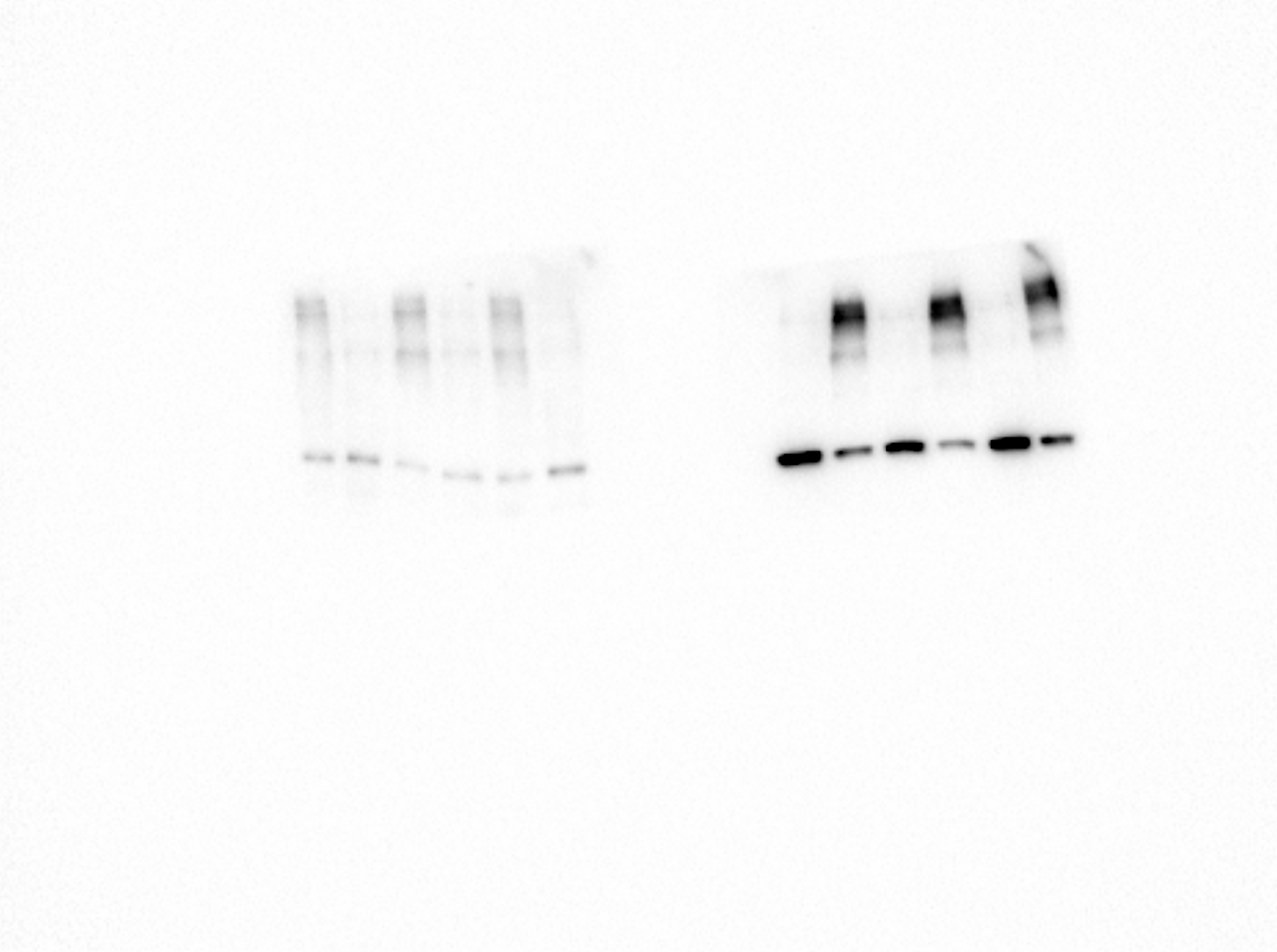

Supplement: Figure 4—source data 2. [file elife-76425-fig4-data2.zip › Figure 4 - source data 2/Figure 4C - GluA1 raw image.tif]

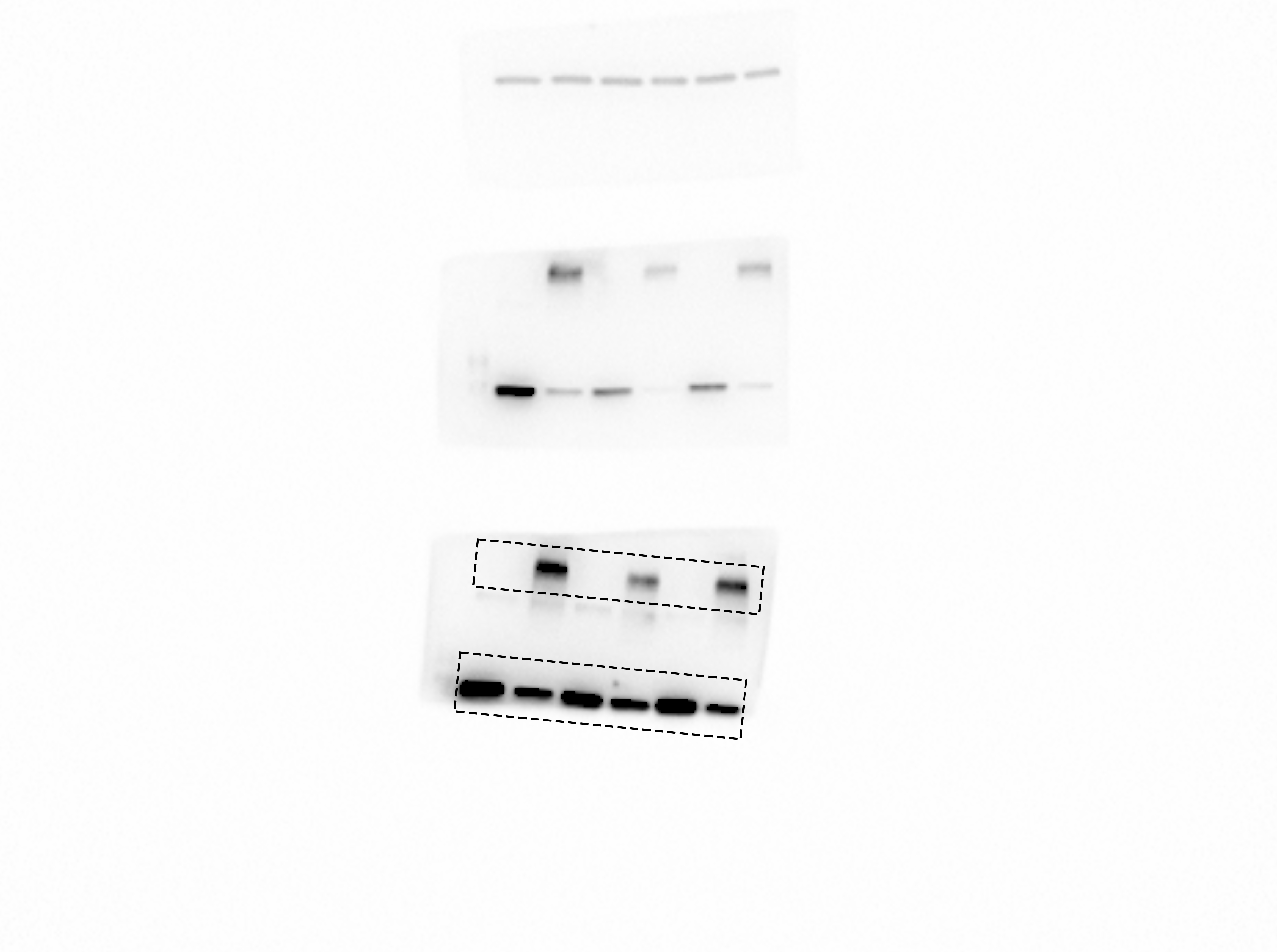

Supplement: Figure 4—source data 2. [file elife-76425-fig4-data2.zip › Figure 4 - source data 2/Figure 4C - GluA23 blot with cropped area.jpg]

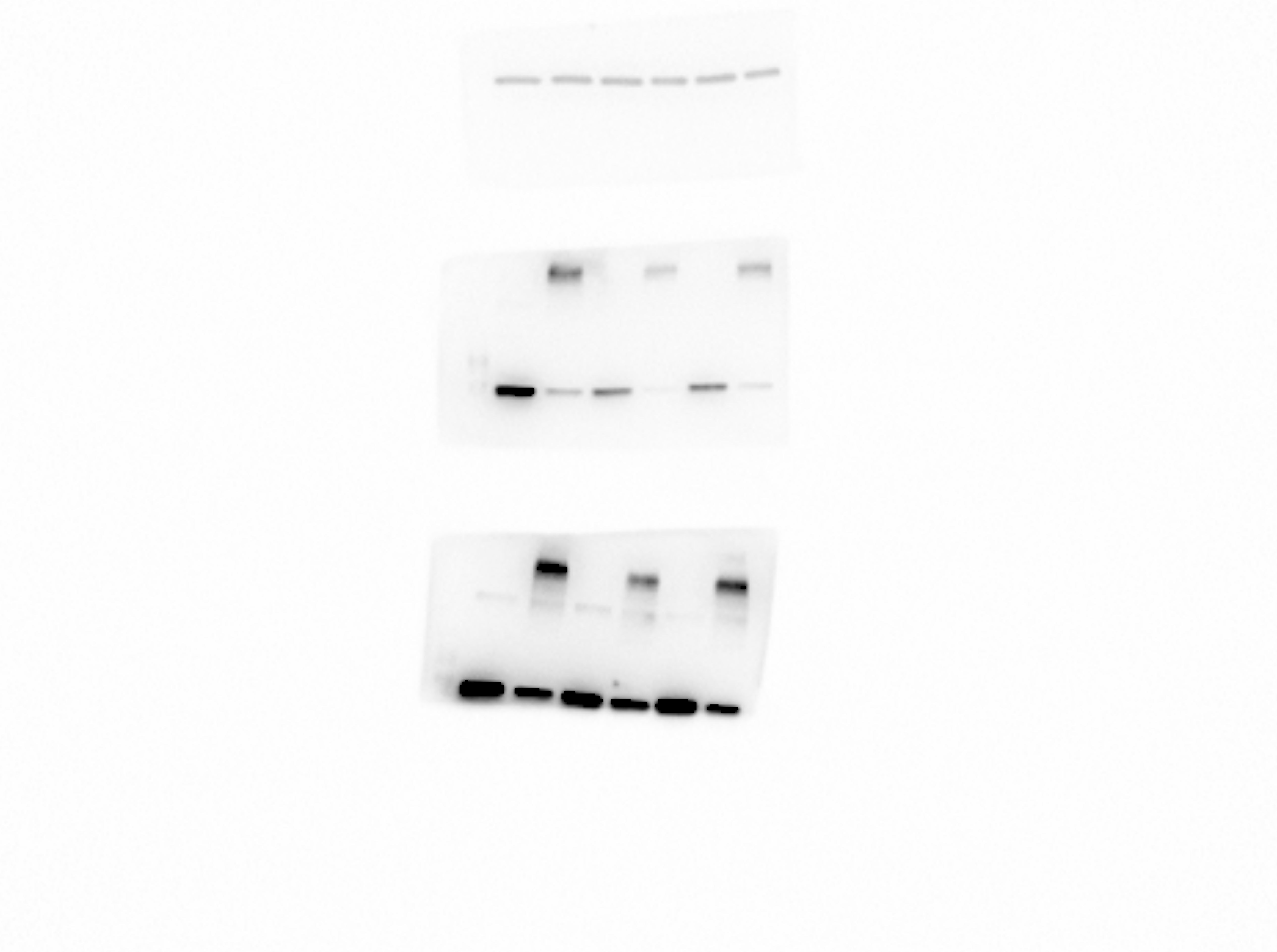

Supplement: Figure 4—source data 2. [file elife-76425-fig4-data2.zip › Figure 4 - source data 2/Figure 4C - GluA23 raw image.tif]

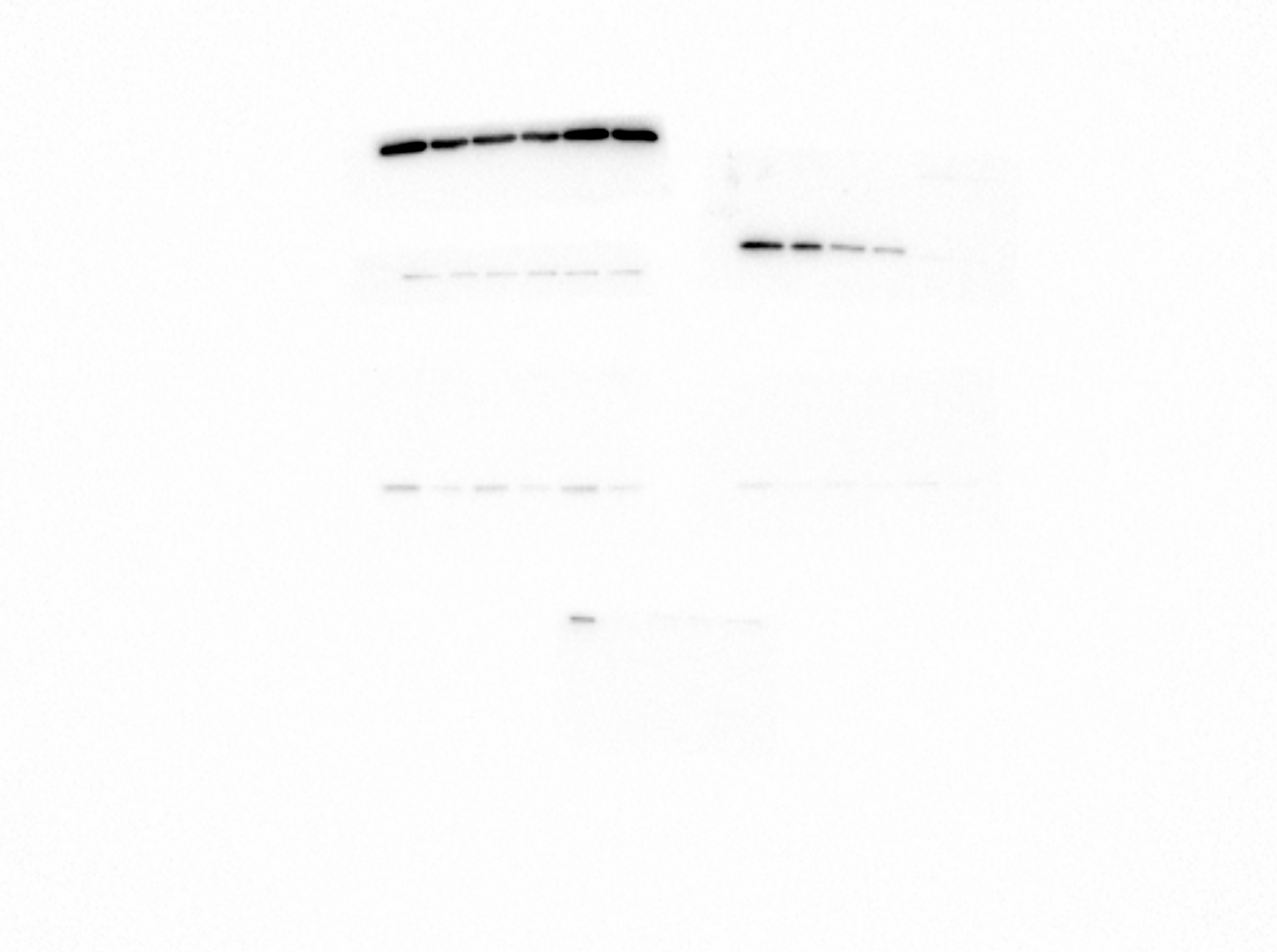

Supplement: Figure 4—source data 2. [file elife-76425-fig4-data2.zip › Figure 4 - source data 2/Figure 4C - Tubulin (GluA1) blot raw data.tif]

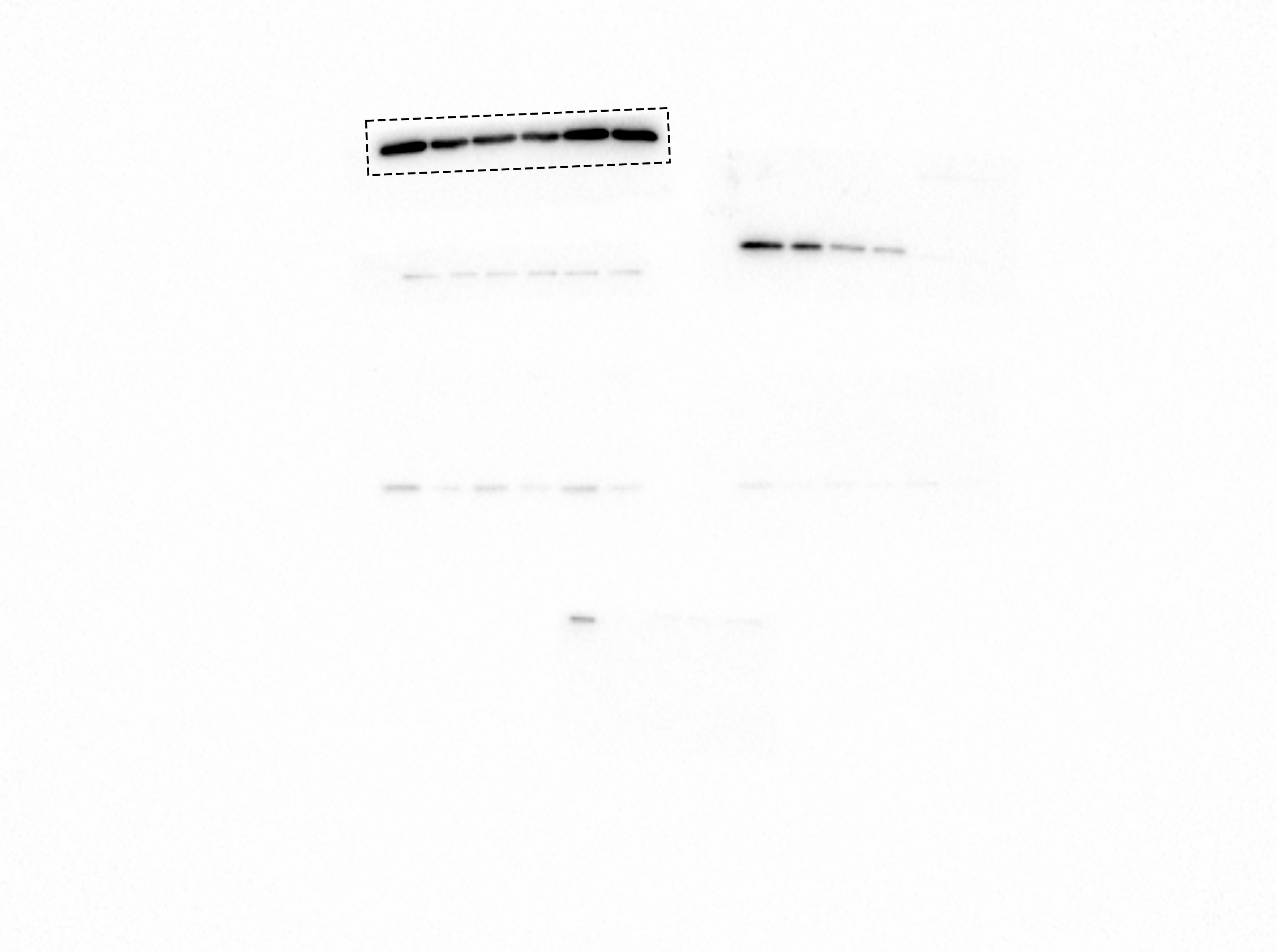

Supplement: Figure 4—source data 2. [file elife-76425-fig4-data2.zip › Figure 4 - source data 2/Figure 4C - Tubulin (GluA1) blot with cropped area.jpg]

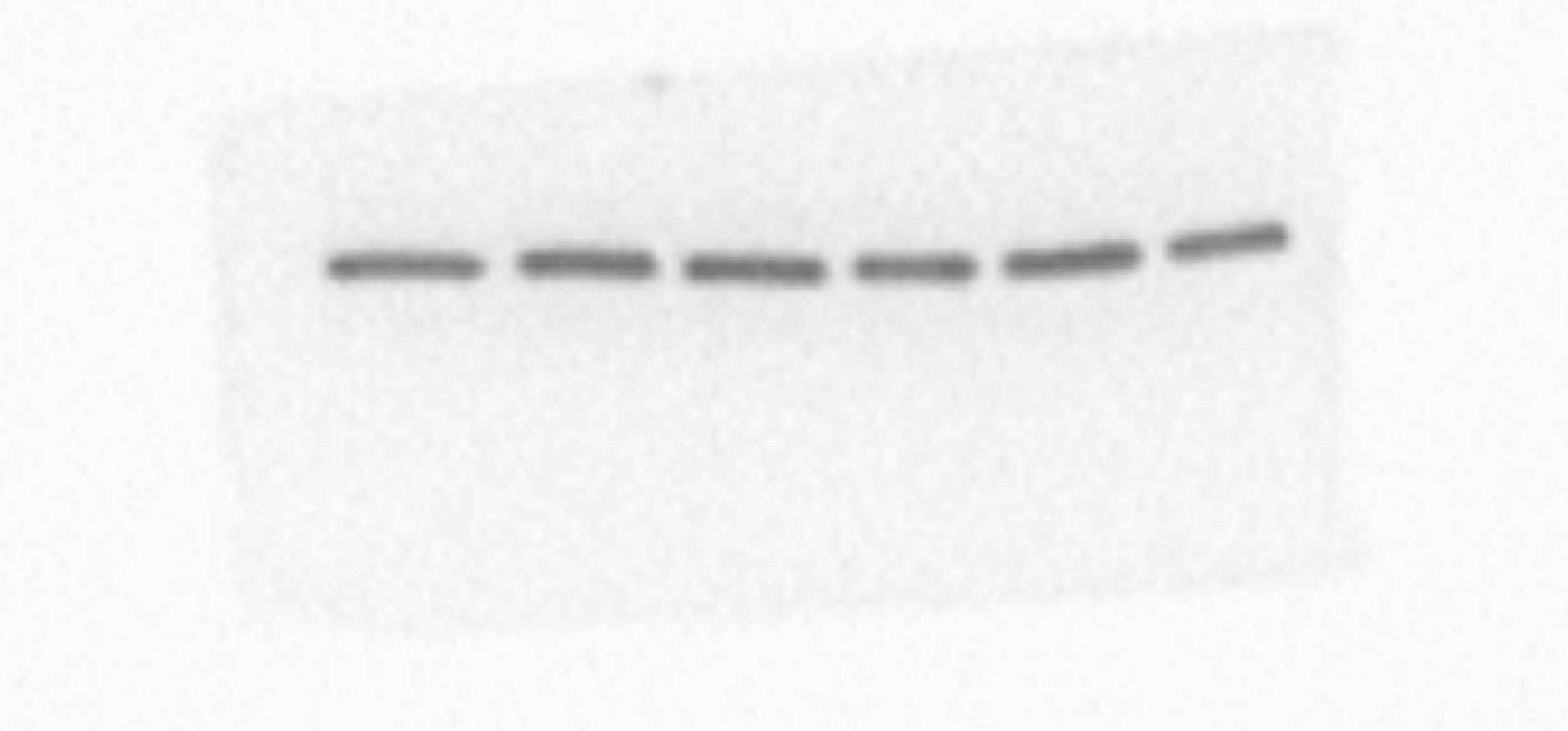

Supplement: Figure 4—source data 2. [file elife-76425-fig4-data2.zip › Figure 4 - source data 2/Figure 4C - Tubulin (GluA23) blot raw data.jpg]

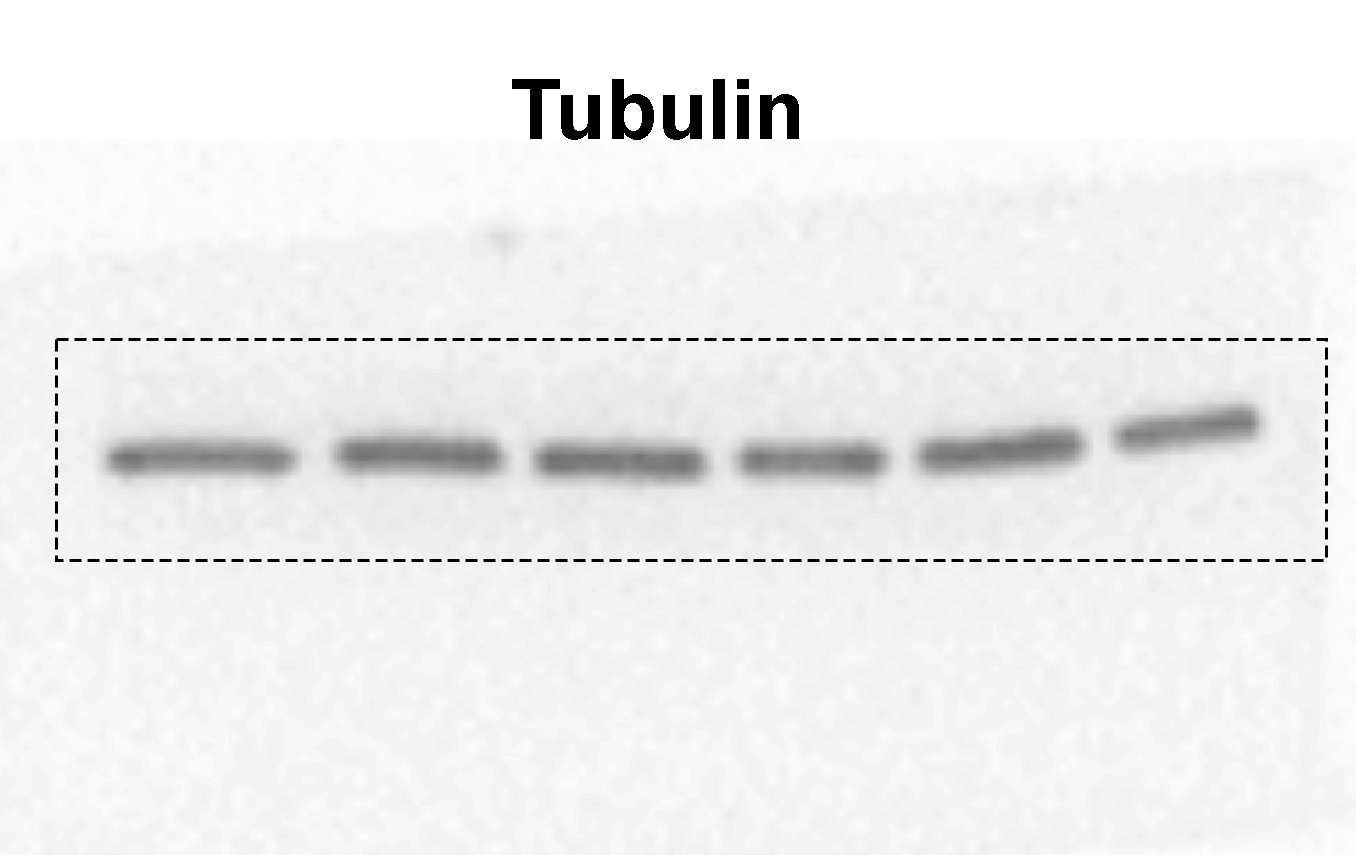

Supplement: Figure 4—source data 2. [file elife-76425-fig4-data2.zip › Figure 4 - source data 2/Figure 4C - Tubulin (GluA23) blot with cropped area.jpg]

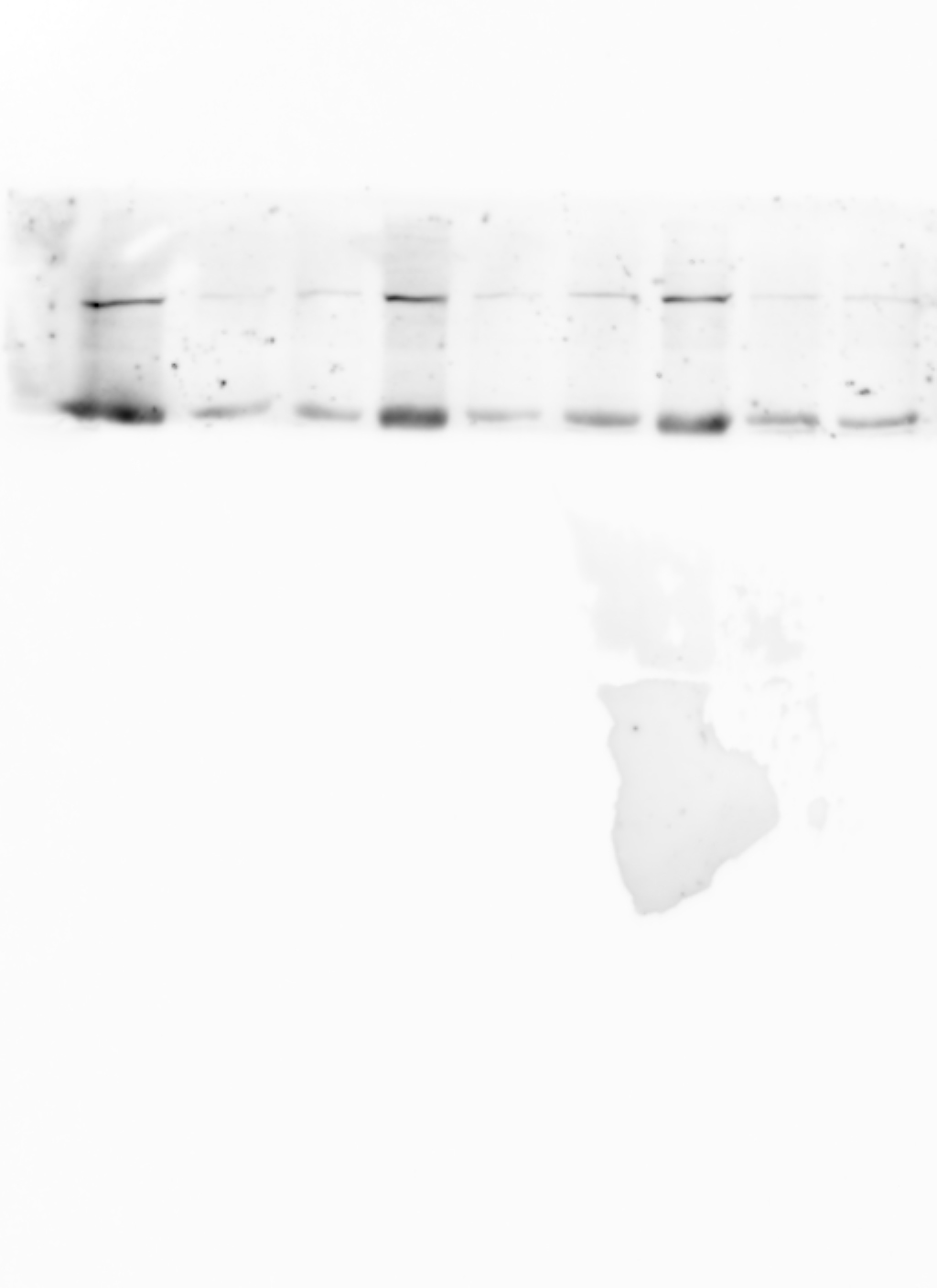

Supplement: Figure 5—figure supplement 1—source data 2. [file elife-76425-fig5-figsupp1-data2.zip › Figure 5 - Figure supplement 1 - source data 2/Panel B - AP4E blot raw image.jpg]

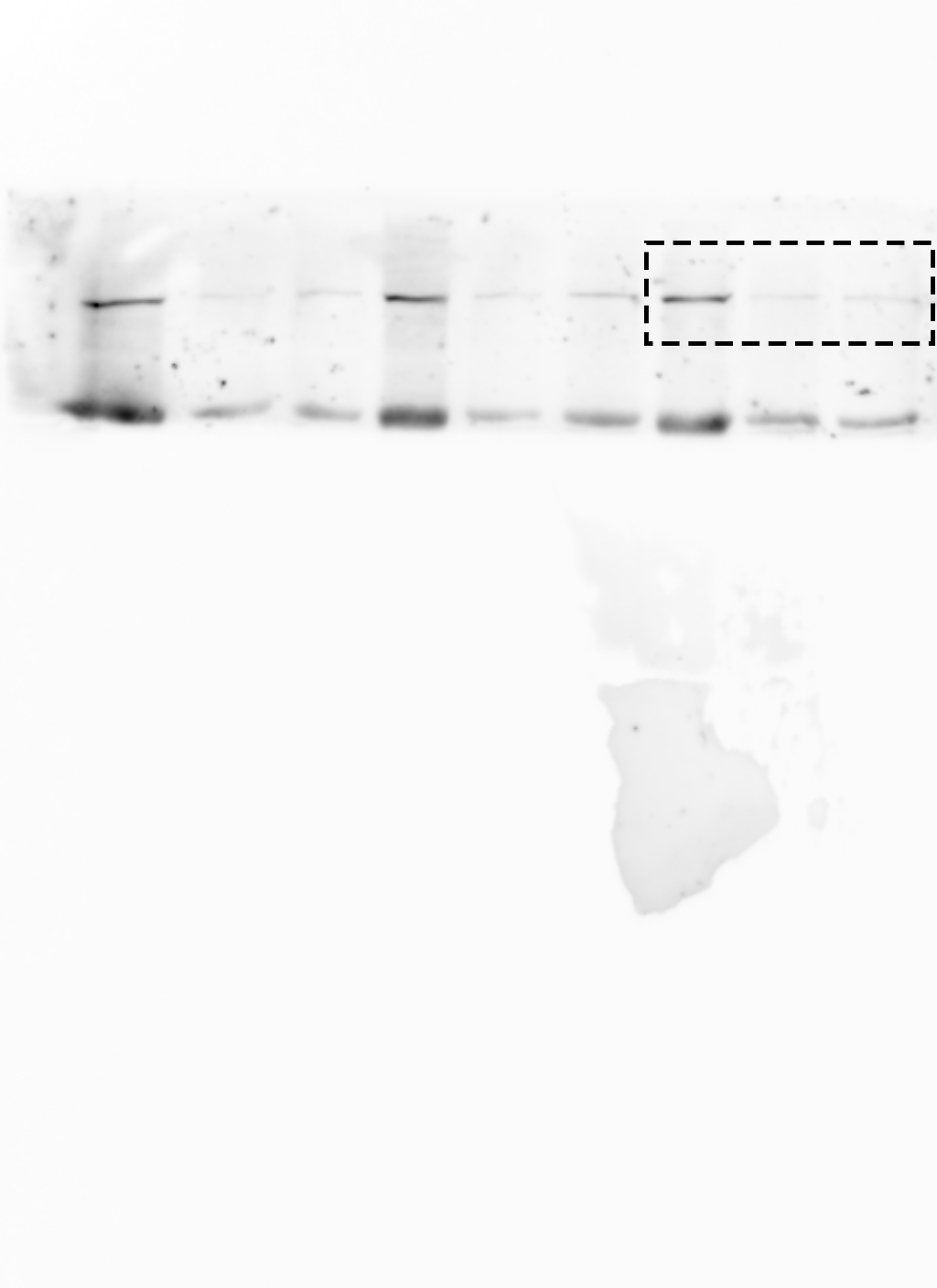

Supplement: Figure 5—figure supplement 1—source data 2. [file elife-76425-fig5-figsupp1-data2.zip › Figure 5 - Figure supplement 1 - source data 2/Panel B - AP4E blot with cropped area.jpg]

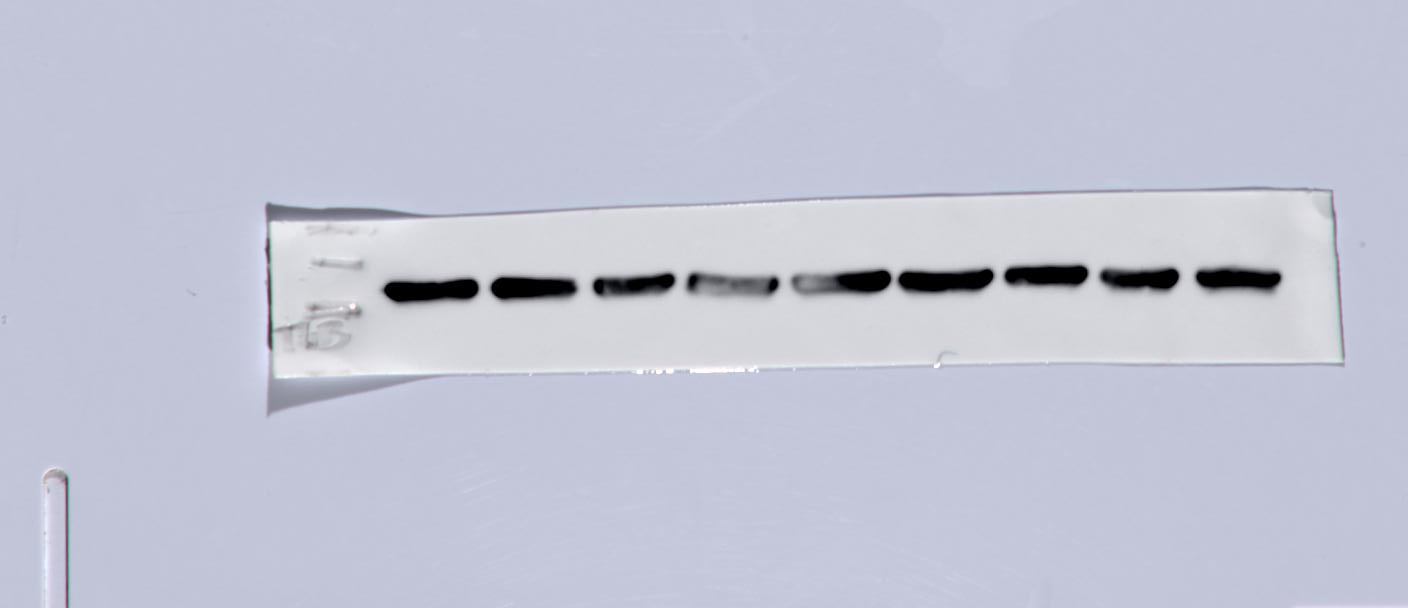

Supplement: Figure 5—figure supplement 1—source data 2. [file elife-76425-fig5-figsupp1-data2.zip › Figure 5 - Figure supplement 1 - source data 2/Panel B - beta3tubulin blot raw image.jpg]

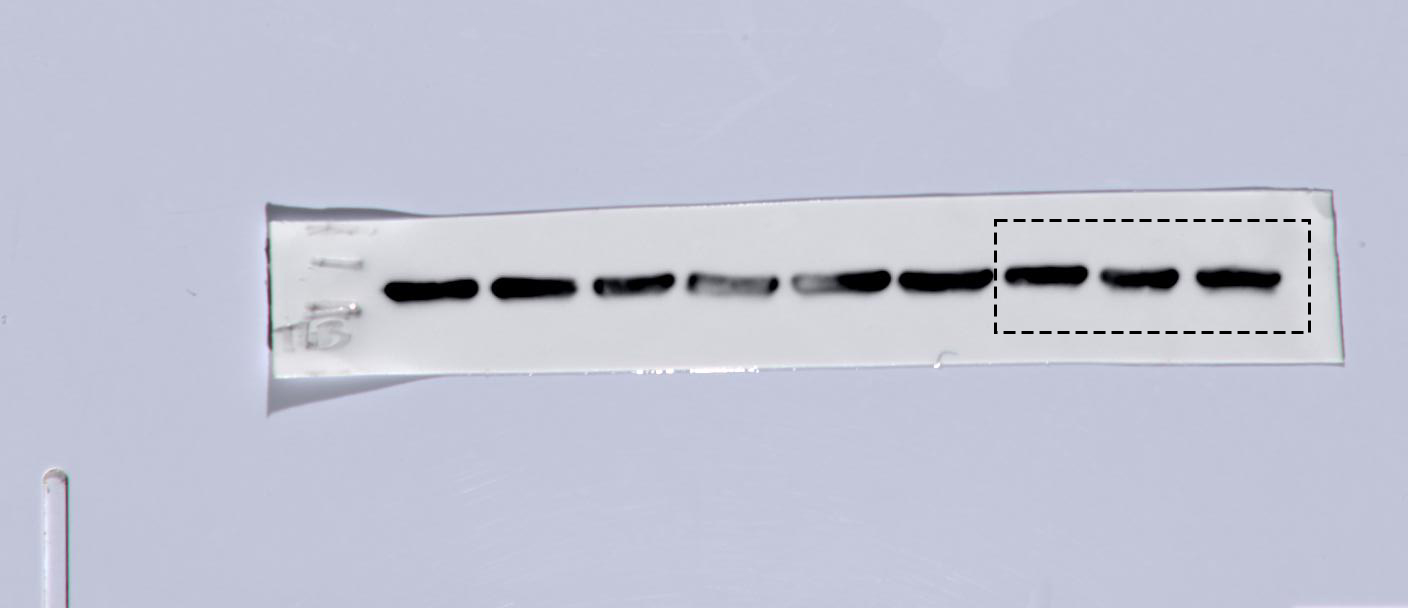

Supplement: Figure 5—figure supplement 1—source data 2. [file elife-76425-fig5-figsupp1-data2.zip › Figure 5 - Figure supplement 1 - source data 2/Panel B - beta3tubulin blot with cropped area.jpg]

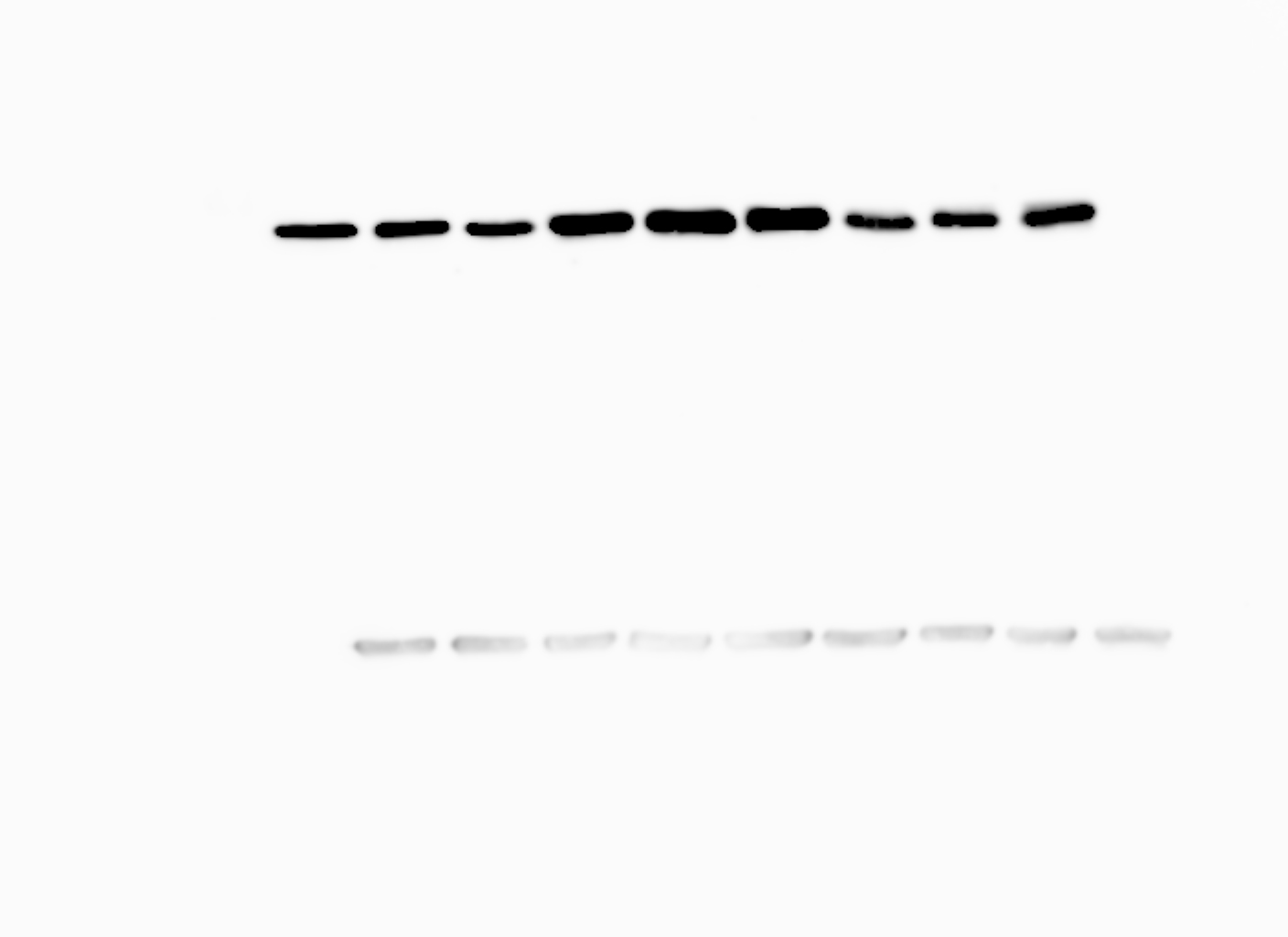

Supplement: Figure 5—figure supplement 1—source data 2. [file elife-76425-fig5-figsupp1-data2.zip › Figure 5 - Figure supplement 1 - source data 2/Panel B - GFP blot raw image.jpg]

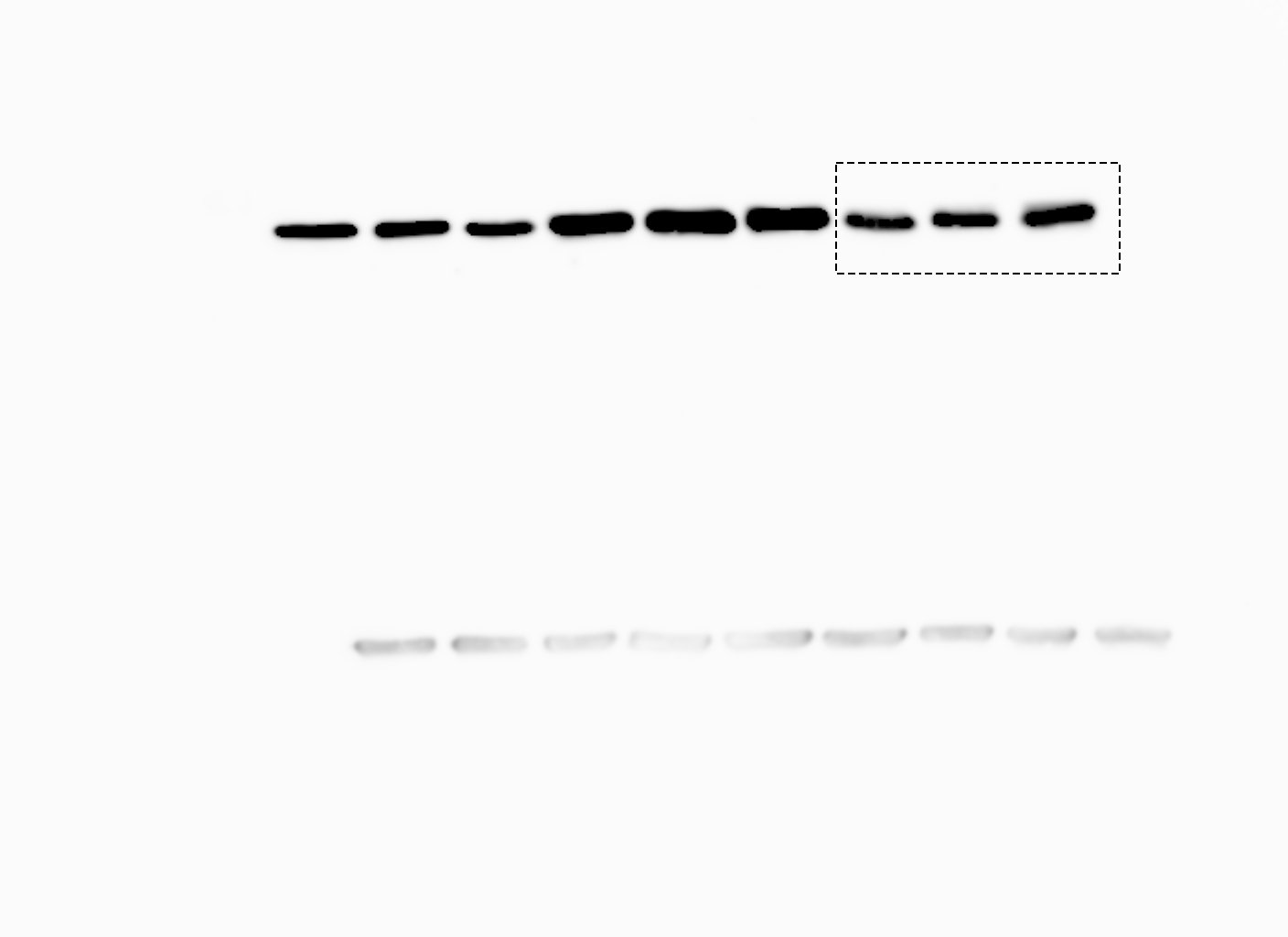

Supplement: Figure 5—figure supplement 1—source data 2. [file elife-76425-fig5-figsupp1-data2.zip › Figure 5 - Figure supplement 1 - source data 2/Panel B - GFP blot with cropped area.jpg]
